# Two-step generation of mesenchymal stem/stromal cells from human pluripotent stem cells with reinforced efficacy upon osteoarthritis rabbits by HA hydrogel

**DOI:** 10.1186/s13578-020-00516-x

**Published:** 2021-01-06

**Authors:** Leisheng Zhang, Yimeng Wei, Ying Chi, Dengke Liu, Sijun Yang, Zhongchao Han, Zongjin Li

**Affiliations:** 1 The Postdoctoral Research Station, School of Medicine, Nankai University, 94 Weijin Road, Tianjin, 300071 China; 2 The Enterprise Postdoctoral Working Station, Tianjin Chase Sun Pharmaceutical Co., Ltd, Tianjin, 301700 China; 3 Department of Neurosurgery, The First Affiliated Hospital of Shandong First Medical University, Ji-nan, 250014 China; 4 Precision Medicine Division, Health-Biotech (Tianjin) Stem Cell Research Institute Co., Ltd, Tianjin, 301700 China; 5 State Key Laboratory of Experimental Hematology & National Clinical Research Center for Blood Disease, Institute of Hematology & Blood Diseases Hospital, Chinese Academy of Medical Sciences & Peking Union Medical College, Tianjin, 300020 China; 6 Jiangxi Research Center of Stem Cell Engineering, Jiangxi Health-Biotech Stem Cell Technology Co., Ltd, Shangrao, 334000 China

**Keywords:** hPSC-MSCs, Programming, Genetic variation, Immunoregulation, Osteoarthritis, HA hydrogel

## Abstract

**Background:**

Current studies have enlightened the rosy prospects of human pluripotent stem cell (hPSC)-derived mesenchymal stem/stromal cells (MSCs) in regenerative medicine. However, systematic investigation of their signatures and applications with alternative biomaterials in osteoarthritis (OA) remains indistinct.

**Methods:**

Herein, we initially took advantage of a small molecule library-mediated programming strategy for hPSC-MSC induction. Then, with the aid of multifaceted analyses such as flow cytometry (FCM), chromosome karyocyte and cell vitality, wound healing and microtubule formation assay and coculturing with T lymphocytes, we systematically evaluated the characterizations of signatures in vitro and the in vivo efficacy of hPSC-MSCs and HA hydrogel composite on rabbit osteoarthritis model.

**Results:**

We found the combination of LLY-507 and AZD5153 was sufficient for high-efficiency CD73^+^CD90^+^CD105^+^CD31^−^CD34^−^CD45^−^HLA-DR^−^ MSC induction from both hESCs and hiPSCs with stemness (*POU5F1*/*SOX2*/*NANOG*). The programmed hPSC-MSCs revealed conservative transcriptome variations and went through a heterogeneous intermediate-stage with mesenchymal-associated gene expression (*NT5E*, *ENG*, *VIM* and *FN1*) as well as displayed typical cytomorphology, immunophenotypes and normal karyotyping, multilineage differentiation potential, favorable cell vitality, proangiogenic and immunoregulatory properties in vitro. Meanwhile, the cell population exhibited preferable restorative and ameliorative function on OA rabbits with HA hydrogel in vivo.

**Conclusions:**

Collectively, we established a rapid and convenient procedure for hPSC-MSC generation without redundant manipulations. The fundamental and clinical studies upon osteoarthritis (OA) treatment would benefit tremendously from the combination of the inexhaustible hPSC-MSCs and advantageous biomaterials.

## Background

Mesenchymal stem/stromal cells (MSCs), acknowledged as multipotent mesenchymal progenitor/precursor cells, medicinal signaling cells or skeletal stem cells, are heterogeneous populations with hematopoietic supporting and immunoregulatory attributes as well as multilineage differentiation potential [1–3]. We and other investigators have verified the dominant role of MSCs in multiple physiological and pathological microenvironments, together with clinical and preclinical applications in regenerative medicine such as osteoarthritis [4], aplastic anemia [5, 6], Crohn’s disease [7], immune thrombocytopenia [8], fulminant hepatic failure [9], acute myocardial infarction (AMI) [10], graft-versus-host disease (GVHD) [11] and even the current corona virus disease 2019 (COVID-19) [12] through direct- and trans-differentiation, autocrine and paracrine, targeting immune-regulating cells and repairing damaged tissues. Since the 1960s, MSCs have been successfully isolated and identified from extensive sources including adult tissues (bone marrow, dental pulp, adipose and liver tissue) and perinatal tissues (placenta, umbilical cord, amniotic fluid and membrane) [13–16]. Among them, the bone marrow-derived BM-MSCs and umbilical cord-derived UC-MSCs are most recognized with the multiple applications and long-term proliferation properties, respectively [5, 11, 17]. However, even though with a variety of superiorities, the tissue-derived MSCs simultaneously have some general concerns before large-scale applications such as declined long-term in vitro proliferation, individual variations in quantity and quality, invasive procedures, pathogenic and ethical risks, which further failed on the assessments of MSCs upon safety, effectiveness and repeatability [18–20].

State-of-the-art updates targeting on fundamental and clinical studies of human pluripotent stem cells (hPSCs) have prompted the prospects and feasibility of alternative MSC generation for regenerative medicine including human induced PSCs (hiPSCs) and embryonic stem cells (hESCs) [20, 21]. To date, numerous randomized prospective reports have suggested the eutherapeutic and ameliorative effect on refractory and recurrent disease remodeling [22, 23]. For instance, Soontararak and his colleagues demonstrated the preclinical equivalence of hiPSC-MSCs and adipose-derived AD-MSCs in promoting microbiome normalization and intestinal healing during inflammatory bowel disease [24]. Similarly, We and Wang et al.verified the therapeutic effect of hESC-MSCs on acute colitis and experimental autoimmune encephalomyelitis (EAE) of multiple sclerosis model, respectively [21, 25, 26]. In spite of the considerable progress in hPSC-MSCs with multifaceted advantages including the infinite proliferation potential, no ethical risks, homogeneity and illimitation in supply, yet most of the current monolayer, coculture and triaxial embryoid body (EB) procedures were accompanied with substantial drawbacks such as time-consuming with low-efficiency, cumbersome manipulations or even lentivirus-dependent programming, which further hinder the in vitro developmental process and regulatory mechanism studies of MSCs as well [21, 27, 28].

In this study, we took advantage of a programming strategy without gene-editing based on the monolayer model and a commercialized small molecule library for high-efficiency hPSC-MSC generation within two weeks. On the one hand, the derived hPSC-MSCs revealed conservative variations in transcriptome and satisfied the essential criteria including typical cytomorphology, immunophenotypes, and multidirectional differentiation potential, together with multidimensional characterizations such as favorable cellular vitality, chromosomal stability, proangiogenic potential and immunosuppressive property in vitro. On the other hand, combined with a unique HA hydrogel biomaterial, the in vivo therapeutic and alleviative effect of hESC-MSCs on osteoarthritis model were further reinforced and manifested whereas the cell vitality was minimally affected as well. Taken together, we have established a convenient and high-efficient procedure for large-scale hPSC-MSC generation with prospects in fundamental research and regenerative medicine, especially for reinforced osteoarthritis administration aided by HA hydrogel.

## Results

### Establishment of a two-step strategy for high-efficiency generation of MSCs from hPSCs

**Fig. 1:**
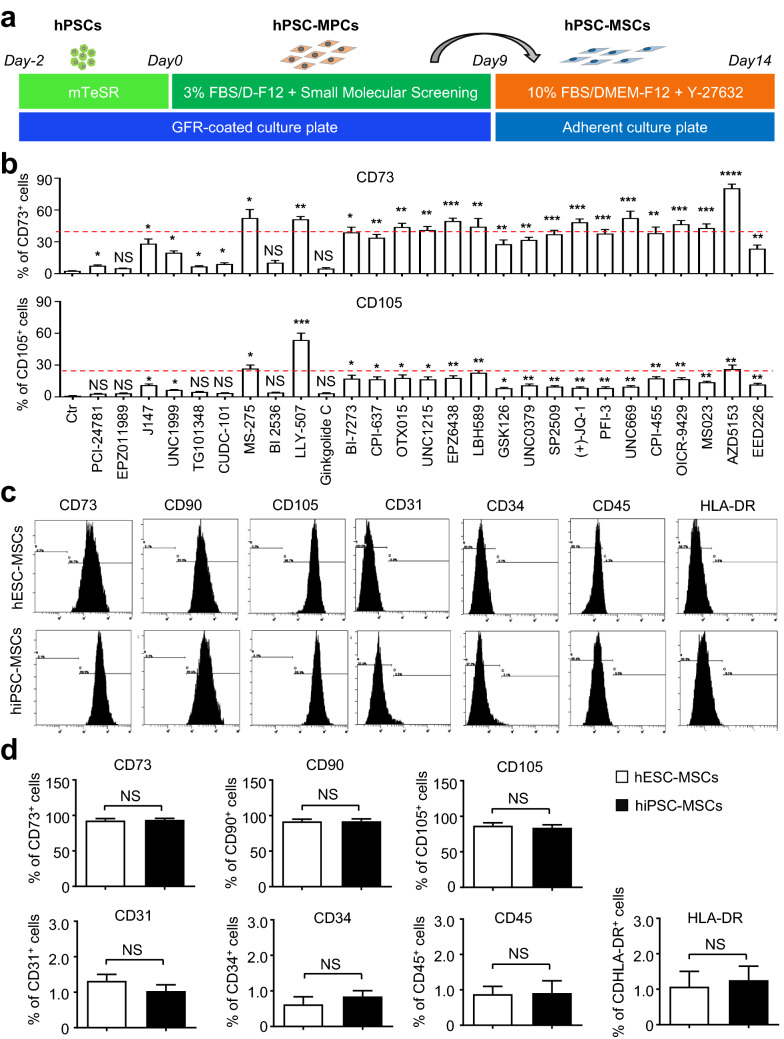
The establishment of a high-efficiency procedure for hPSC-MSCs generation. **a** Schematic illustration of the procedure for hESC-MSCs induction. **b** FCM analysis of CD73^+^ and CD105^+^ hESCs-derived cells at days 9. **c** Flow cytometry (FCM) analysis of MSC-associated biomarkers in hESC-MSCs. **d** Statistic analysis of the aforementioned biomarkers in hPSC-MSCs. All data were shown as mean ± SEM (N = 3). *P < 0.05, **P < 0.01, ***P < 0.001, ****P < 0.0001; NS, not significant

Over the years, we and other investigators have demonstrated hPSC-MSCs as splendid alternative and controllable sources for cytotherapy in regenerative medicine [20, 21, 23]. Nevertheless, establishment of a high-efficiency and cost-effective procedure for homogeneous hPSC-MSC generation is the prerequisite for large-scale clinical applications [22]. For the purpose, we took advantage of a small molecule-mediated “two-step” programming strategy as we recently reported with minimal modifications [21, 22] (Fig. 1a). Incipiently, as described in the Methods, 2–3 × 10^4^/ml H1 hESCs were seeded on GFR-coated 6-well plate in E8 medium for two days, then the medium was changed into 3% FBS/DMEM-F12 medium with/without 10 nM small molecule addition. The percentage of generated MSC-like progenitor cells (hESC-MPCs) was quantified by FCM analysis. Differ from the other small molecules in the commercial library (TargetMol, Shanghai, China), HDAC inhibitors (MS-275, LBH589), PI3K/PLK or JAK/STAT or BRD4 inhibitors (LLY-507, OICR-9429, AZD5153), epigenetic reader domain inhibitors (OTX015, UNC1215, (+)-JQ-1, UNC669, OICR-9429, MS023, AZD5153, EPZ6438), histone methyltransferase (H3K9me and H3K27me)-associated PCR2/EED-EZH2 complex inhibitor (EPZ6438), and in particular, AZD5153 addition could significantly enhanced CD73^+^ hESC-MPC generation with a proportion of over 40% (Fig. 1b, Additional file 1: Figure S1a). As for the CD105^+^ population, only MS-275, LLY-507 or AZD5153 addition could simultaneously induce over 25% of hESC-MPC generation (Fig. 1b, Additional file 1: Figure S1a). Thereafter, aiming to further enhance hESC-MPC generation and facilitate the more mature CD73^+^CD105^+^ hESC-MSC induction, we combined LLY-507 with AZD5153 and found that nearly 90% of H1 hESC-derived cells conformed to the standard immunophenotypes of MSCs, which was further reinforced with a single passaging (Fig. 1c, d). Similarly, with the combination of the aforementioned LLY-507 with AZD5153, a comparable proportion of hiPSC-MSCs was obtained as well, which further confirmed that the two-step procedure was competent and convenient for high-efficiency induction of multiple hPSC-MSCs as well (Fig. 1c, d). Taken together, with the aid of small molecule library screening, we verified that simultaneous programming with two specific epigenetic modification- or signaling pathway-related small molecules was sufficient for high-efficiency hESC-MSC generation.

### hPSC-MSCs exhibited favorable characteristics in multilineage differentiation with consistency of karyotyping and proliferation

**Fig. 2:**
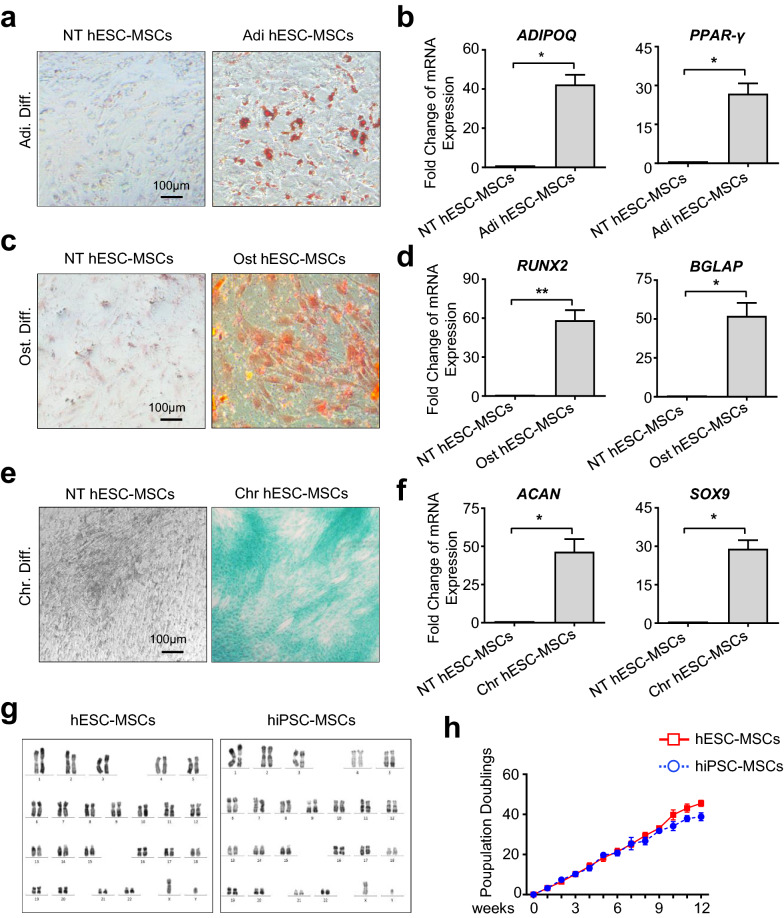
hESC-MSCs exhibit preferable characteristics in multilineage differentiation, karyotyping and proliferation. **a** The phase contrast images of hESC-MSC-derived adipocytes with Oil red O staining. Scale bar = 100 μm. **b** qRT-PCR analysis of the adipogenic-associated genes. **c** Alizarin Red staining of hESC-MSC-derived osteoblasts. Scale bar = 100 μm. **d** qRT-PCR analysis of the osteogenic-associated genes. **e** Alcian Blue staining of hESC-MSC-derived chondrocytes. Scale bar = 100 μm. **f** qRT-PCR analysis of the chondrogenic-associated genes. **g** Karyotypic analysis of hPSC-MSCs with G-banded chromosome experiment. **h** Long-term in vitro Expansion potential of hPSC-MSCs for 12 passages by Pd assay. All data were shown as mean ± SEM (N = 3). *P < 0.05, **P < 0.01; NS, not significant

To further explore whether the derived hPSC-MSCs were functionally mature, we turned to the multilineage differentiation analysis, which was also the golden standard for MSC identification [21, 23]. Compared with the undifferentiated hPSC-MSCs (NT hESC-MSC, NT hiPSC-MSC), substantial adipocytes were generated and dyed for Oil Red O staining in the differentiated groups (hESC-MSC, hiPSC-MSC) after a 2-week’s adipogenic differentiation, which were further confirmed by qRT-PCR analysis of *ADIPOQ* and *PPAR-γ* (Fig. 2a, b, Additional file 2: Figure S2a, b). Similarly, as shown by the Alizarin Red S staining of hPSC-MSC-derived osteoblasts and quantitative analysis of *RUNX2* and *BGLAP* expression, the differentiated hESC-MSC and hiPSC-MSC at day 18 exhibited comparable osteogenic differentiation capacity (Fig. 2c, d, Additional file 2: Figure S2c, d). With the aid of Alcian Blue staining and detection of representative cartilage-associated genes including *ACAN* and *SOX9*, we found that the indicated two kind of hPSC-MSCs showed distinguishable chondrogenic differentiation potential as well (Fig. 2e, f, Additional file 2: Figure S2e, f). Hence, the small molecule (LLY-507 and AZD5153)-programmed hPSC-MSCs collectively satisfied the fundamental criteria for defining MSCs with typical immunophenotypes and standard multilineage differentiation potential. Meanwhile, by utilizing the G-banded karyotyping analysis we ascertained that the prepared hPSC-MSCs displayed normal chromosome without evident abnormalities as we recently reported [22] (Fig. 2g). In consistence with hESC-MSCs, hiPSC-MSCs showed superiorly and indistinguishably long-term proliferative potential in 10% FBS/DMEM-F12 medium (Fig. 2h).

### hESC-MSCs and hiPSC-MSCs manifested conservative transcriptome variations

**Fig. 3:**
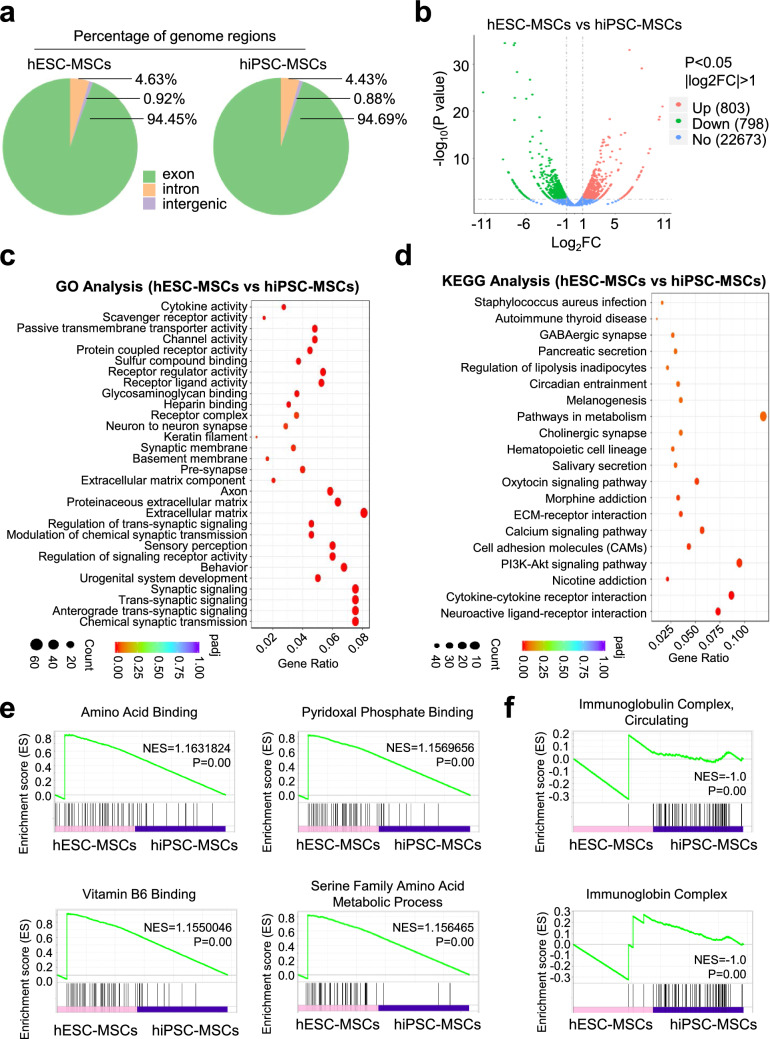
The transcriptome analysis of hESC-MSCs and hiPSC-MSCs. **a** The percentage of the subtypes of genome regions in hESC-MSCs and hiPSC-MSCs, including exon, intron and intergenic. **b** Volcano plot diagram of gene expression profiling between hESC-MSCs and hiPSC-MSCs. **c**, **d** Gene ontology (GO) (**c**) and KEGG (**d**) analysis of differentially expressed genes (DEGs) in hESC-MSCs and hiPSC-MSCs. **e**, **f** GSEA analysis of significantly upregulated DEGs in hESC-MSCs (**e**) and hiPSC-MSCs (**f**), respectively

For the purpose of exploring the potentially genetic variations at transcriptome level, we turned to RNA-SEQ analysis of the programmed hESC-MSCs and hiPSC-MSCs. Generally, both of the hPSC-MSCs revealed similarities in the contents of genome regions, including exon, intron and intergenic (Fig. 3a). As shown by the volcano plot diagram, of the 24,272 genes, a total number of 803 and 798 genes were significantly upregulated and downregulated in hESC-MSCs compared with those in hiPSC-MSCs, respectively (Fig. 3b). Furthermore, we found the differentially expressed genes (DEGs) were involved in multiple biological processes such as extracellular matrix, receptor ligand activity and chemical synaptic transmission (Fig. 3c). Simultaneously, according to the Kyoto encyclopedia of genes and genomes (KEGG) analysis, a series of signaling pathways were enriched including PI3K-Akt, ECM-receptor interaction and pathways in metabolism (Fig. 3d). In consistence with the GO and KEGG analyses, DEGs associated with amino acid binding, pyridoxal phosphate binding, vitamin B6 binding and serine family amino acid metabolic process were collectively upregulated in hESC-MSCs. Instead, those upregulated DEGs in hiPSC-MSCs were related to immunoglobulin complex, circulating and immunoglobin complex (Fig. 3e, f).

### hPSC-MSC generation through an intermediate stage with a hierarchical spectrum of gene expression

**Fig. 4:**
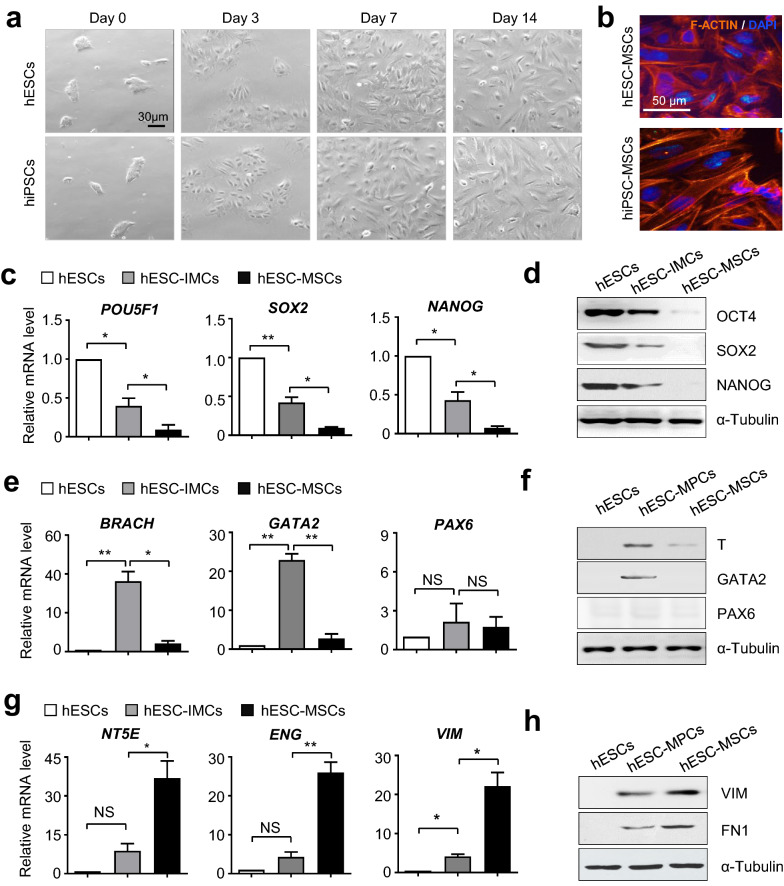
MSCs derived from hESCs through a heterogeneous intermediate stage. **a** Dynamic morphology of derived hPSC-MSCs. Scale bar = 20 μm. **b** Representative immunofluorescence images of β-tubulin in hPSC-MSCs. Scale bar = 50 μm. **c**, **d** qRT-PCR (**c**) and western-blotting (**d**) analyses of pluripotency-associated gene expression in hESCs and derived cells. **e**–**f** qRT-PCR (**e**) and western-blotting (**f**) analyses of germ layer-associated gene expression in hESCs and derived cells. **g**, **h** qRT-PCR (**g**) and western-blotting (**h**) analyses of MSC-associated gene expression in hESCs and derived cells. All data were shown as mean ± SEM (N = 3). *P < 0.05, **P < 0.01; NS, not significant

Having verified the programming strategy for high-efficiency hPSC-MSC induction, we are curious about the particulars during hPSC-MSC differentiation. For the purpose, we initially observed the dynamically morphological changes from small compact colonies into unconsolidated and spindle-like cell population (Fig. 4a, b). In consistence, quantitative analysis revealed that the expression levels of pluripotency-associated genes, including *POU5F1* (also known as OCT4), *SOX2* and *NANOG*), in hPSC-MSCs (hESC-MSCs, hiPSC-MSCs) were sharply downregulated when compared with those in the undifferentiated hPSCs, which indicated the differentiation process (Fig. 4c, d, Additional file 3: Figure S3a). Simultaneously, we found the instantaneous upregulation of multiple germ layer-associated genes both at the mRNA and protein levels during hPSC-MSC generation, including *BRACHARY* (also known as *T*), *GATA2* and *PAX6*, which were involved in mesoderm, endoderm and ectoderm specification, respectively (Fig. 4e, f, Additional file 3: Figure S3b). Furthermore, detection of representative MSC markers such as *NT5E* (also known as CD73), *ENG* (also known as CD105) and *VIM* displayed the dramatical and progressive elevation in hPSC-MSCs (Fig. 4g, h, Additional file 3: Figure S3c). Taken together, by examining the expression spectrum of indicated genes, we further revealed a heterogeneous intermediate stage during hPSC-MSC development.

### *hPSC-MSCs displayed multifaceted *in vitro* attributes in cytological signatures*

**Fig. 5:**
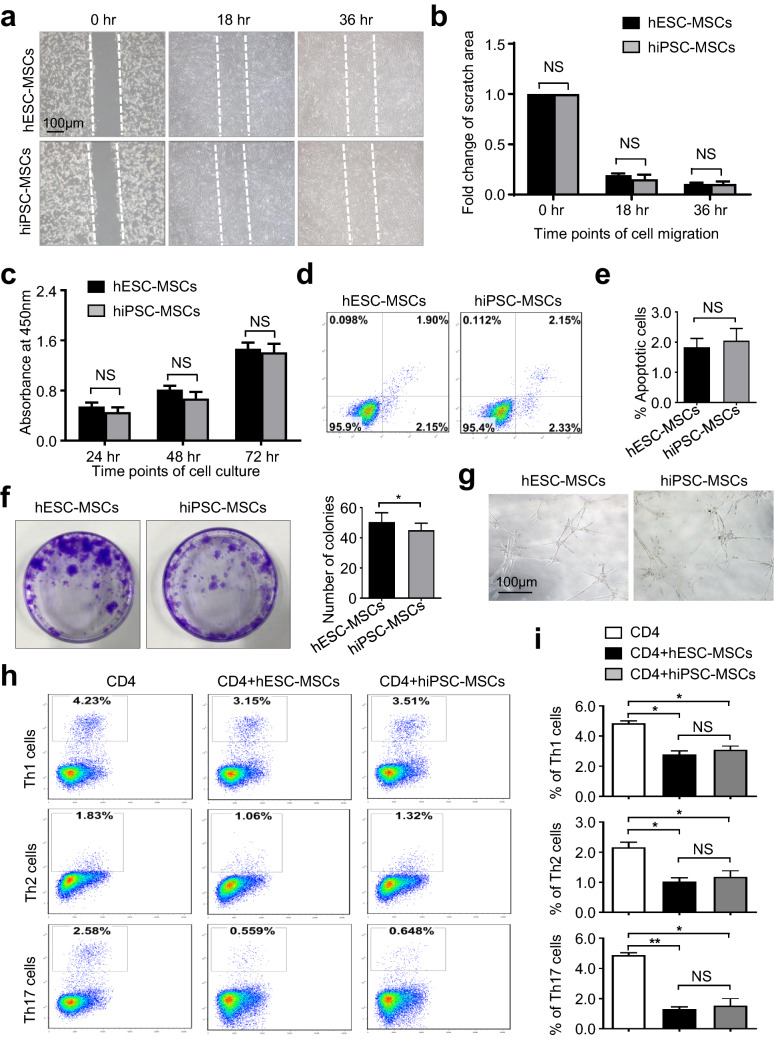
hPSC-MSCs exhibited consistently multifaceted cell vitality and immunoregulatory attributes. **a**, **b** Representative phase contrast images (**a**) and statistical analysis (**b**) of cell migration upon hPSC-MSC. Scale bar = 100 μm. **c** CCK-8 analysis of hPSC-MSCs during cell culture. **d**, **e** The percentages of apoptotic population in hPSC-MSCs were shown by the FCM diagram (**d**) and statistical analysis (mean ± SEM, N = 3) (**e**). **f** CFU-F analysis of the seeded hPSC-MSCs. **g** Microtubule formation assay of total tube number and length by hPSC-MSCs. **h**, **i** FCM analysis and statistical analysis of the differentiated Th1, Th2 and Th17 cells. All data were shown as mean ± SEM (N = 3). *P < 0.05, **P < 0.01; NS, not significant

Of the reported parameters, cell homing and vitality have been acknowledged as the dominating matters for the quality and long-term effectiveness of MSCs on efficacy of diseases both in preclinical and clinical trials [22, 29]. Hence, we originally conducted wound healing analysis to test the homing-associated migration capacity of hPSC-MSCs (hESC-MSCs and hiPSC-MSCs). Distinguished from the initial time point (0 h), the hPSC-MSCs at 18 h and 36 h were visibly fused, which was confirmed by the statistical analysis of fold change of scratch areas (Fig. 5a, b). Meanwhile, with the aid of a cell counting kit-8 (CCK-8) reagent and flow cytometry analysis, we subsequently found that the indicated hPSC-MSCs showed comparable cell vitality including cell expansion and apoptotic cells (Fig. 5c, e).

At the same time, hESC-MSCs displayed moderate superiority over hiPSC-MSCs in colony formation, which was demonstrated by practicing the standard fibroblast colony-forming unit (CFU-F) assay (Fig. 5f). However, we clarified that both of the indicated hPSC-MSCs exhibited equal proangiogenic potential based on microtubule formation assay with Matrigel (Fig. 5g). Besides, it’s of equal importance to investigate the immunoregulatory attribute of hPSC-MSCs as well. Hence, we took advantage of the coculture model to evaluate the inhibitory effect of hPSC-MSCs on CD4^+^ T lymphocytes isolated from human peripheral blood mononuclear cells (PBMCs) as we recently reported [6, 21]. Unsurprisingly, similar to the hESC-MSCs group, there was merely diacritical differences upon the inhibitory ability of hiPSC-MSCs on activation of CD3^+^ T cells and differentiation towards Th1, Th2 and Th17 were observed (Fig. 5h, i). Collectively, the abovementioned results revealed that both hPSC-MSCs showed satisfactory attributes in multifaceted cytological signatures.

### *HA hydrogels enhanced *in vitro* chondrogenic differentiation of hESC-MSCs whereas minimally affected cell vitality*

**Fig. 6:**
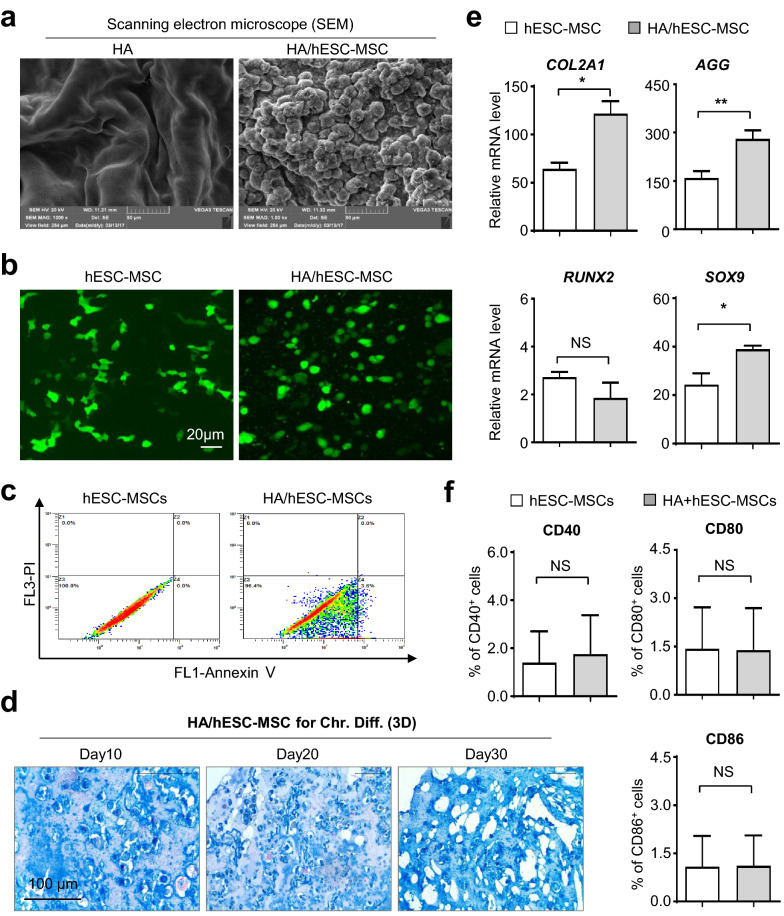
HA hydrogels enhanced in vitro chondrogenic differentiation of hESC-MSCs whereas minimally affected cell vitality. **a** Scanning electron microscope (SEM) analysis of HA hydrogels (HA) and HA/hESC-MSCs hydrogel (HA/hESC-MSCs). **b** Immunofluorescence analysis of F-ACTIN cytoskeleton of monolayer cultured hESC-MSCs and HA/hESC-MSCs. Scale bar = 20 μm. **c** FCM analysis of apoptotic population in hESC-MSCs and HA/hESC-MSCs. **d** The dynamic images of sections of HA/hESC-MSCs-derived chondrocytes with Alcian Blue staining. Scale bar = 100 μm. **e** qRT-PCR analysis of the chondrogenic-associated genes in the hESC-MSCs and HA/hESC-MSCs groups. **f** FCM assay of CD40^+^, CD80^+^ or CD86^+^ cells in hESC-MSCs or HA/hESC-MSCs. All data were shown as mean ± SEM (n = 6). *P < 0.05, **P < 0.01; NS, not significant

In recent years, we and other investigators have indicated the splendid prospects of multitudinous biomaterials for regenerative applications, including the HA hydrogels (HA) by our collaborators, and in particular, for locally refractory disease remodeling [4, 30]. However, the potential impact of HA to signatures and biofunctions of hPSC-MSCs were largely unknown. Considering the minimal distinctions between hESC-MSCs and hiPSC-MSCs, we incipiently utilized the hESC-MSCs for further in vitro and in vivo analysis with HA. With the aid of scanning electron microscope (SEM) technology, we found that the HA/hESC-MSCs composite showed a subjectively botryoidal shape and distinguished from the single HA hydrogels (Fig. 6a). To visualize the details of hESC-MSCs under bidimensional and triaxial circumstances, we conducted immunofluorescent staining of the cytoskeletal F-ACTIN protein and confirmed the cell existence and distribution. Differ from the spindle cytomorphology in monolayer culture, hESC-MSCs showed typical circular form in HA/hESC-MSCs composite instead (Fig. 6b). More importantly, flow cytometry assessment of hESC-MSCs revealed the comparable proportion of apoptotic cell population in the two groups, which manifested the minimal effect of HA on cell vitality (Fig. 6c).

Having clarified the maintenance of cell vitality in HA/hESC-MSCs composite, we were subsequently curious about the potential and dynamic influence towards chondrogenic differentiation in vitro. As shown by the sections of HA/hESC-MSCs composite with Alcian blue staining at the indicated time points, more glycosaminoglycan synthesis was intuitively observed according to the three-dimensional differentiation process (Fig. 6d). Quantitative analysis of the cartilage-associated genes (*COL2A1*, *AGG*, *SOX9*) further confirmed the facilitating effect of HA upon chondrogenic differentiation of hESC-MSCs (Fig. 6e). Meantime, no statistically significant differences in the expression levels of a spectrum of co-stimulatory molecules on hESC-MSCs between the indicated two groups, which congruously indicated the maintenance of hypo-immunogenicity (Fig. 6f, Additional file 4: Figure S4a). Taken together, we came to the conclusion that HA hydrogels could benefit the chondrogenic potential of hESC-MSCs without conspicuous effects on cell vitality.

### HA hydrogels enhanced efficacy of hESC-MSCs on osteoarthritis rabbits

**Fig. 7:**
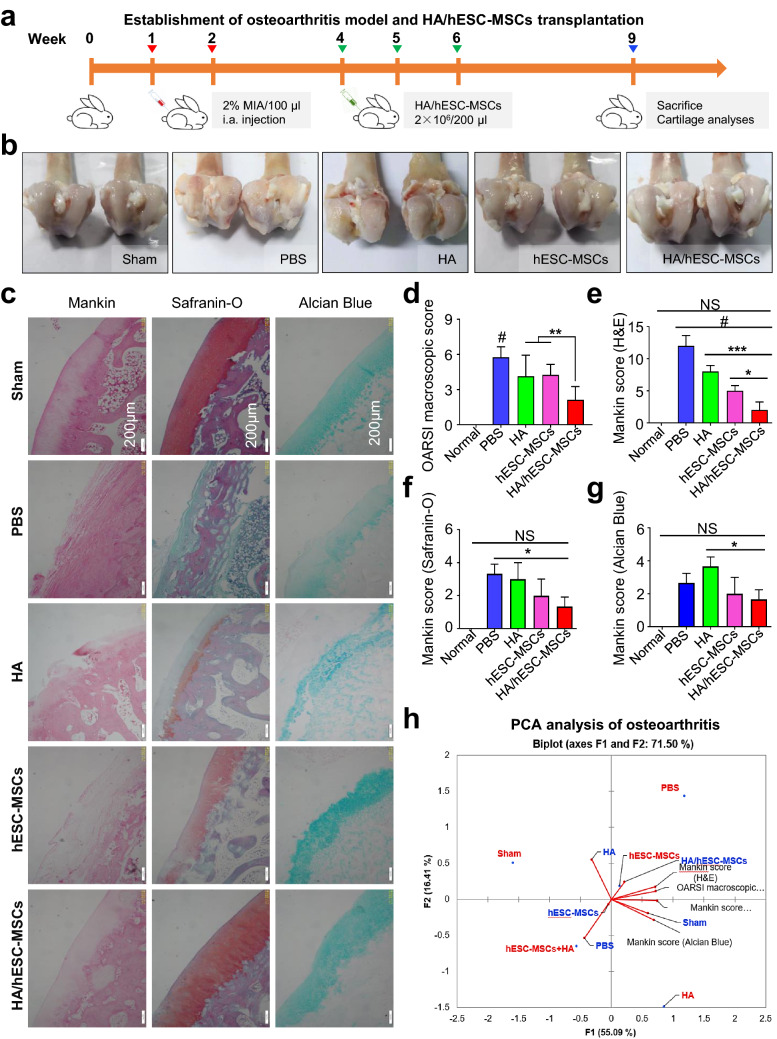
HA hydrogels enhanced in vivo cartilage repair of hESC-MSCs on MIA osteoarthritis mice. **a** Schematic illustration of hESC-MSC and HA hydrogel composite transplantation for cartilage repair of MIA osteoarthritis mice. **b** Clinicopathologic features of osteoarthritis in control mice (Sham) or MIA osteoarthritis mice. **c** The OARSI macroscopic scores of mice in the indicated groups. **d** Microscopic structure of cartilage sections with indicated staining, respectively. **e**–**g** The histopathological scores of cartilage sections in the indicated mice based on the indicated staining, respectively. **h** Illustration of the osteoarthritis-associated parameters as shown in Additional file 5: Figure S5a by PCA (Axes F1 and F2 representing 71.50%). For instance, HA/hESC-MSCs (word labeled in blue) and some variables corresponding to F1 (Mankin score and OARSI macroscopic score) (also see Additional file 5: Figure S5a) showed positive correlation, while HA/hESC-MSCs and Sham were opposed with the PBS (F2) (also see Additional file 5: Figure S5a; dot labeled in blue). All data were shown as mean ± SEM (n = 6). *P < 0.05, **P < 0.01; NS, not significant

To further investigate the potentially curative effect of HA/hESC-MSCs composite in vivo, we turned to the well-established osteoarthritis rabbit model by MIA, which was a rapid and minimally invasive method [3, 4, 31]. Briefly, for the establishment of osteoarthritis model, knee joints of experimental rabbits (thereafter with 1 × PBS or hESC-MSC or HA/hESC-MSC injection, respectively) were treated with 2% MIA for twice on the initially two weeks whereas those without the treatment were served as negative control (Sham). Differ from those in the positive control group (PBS), rabbits were locally injected with hESC-MSC or HA/hESC-MSC (2 × 10^6^ per knee joint) for three times (at week 4, 5, 6). Finally, mice were executed for euthanasia and clinicopathological analysis at week 9 of the model (Fig. 7a). From the general anatomical features of the knee joints, we observed significant alleviation of structural damage in femoral condyles of osteoarthritis rabbits with hESC-MSC administration, and especially those with local HA/hESC-MSC injection, which was further verified by the statistical analysis based on an OARSI macroscopic score (Fig. 7b, c). Besides the aforementioned macroscopic analysis, we further performed histopathologic examinations detections to dissect and compare the similarities and differences in tissue sections of knee joints in rabbits among the indicated groups. With the aid of multifaceted staining, including Mankin, Safranin-O and Alcian Blue, we intuitively and definitively observed more ameliorative disorganizations in rabbits with HA/hESC-MSC application over those merely with hESC-MSC instead (Fig. 7d). Consistently, the more conspicuous improvements in structural and functional characteristics of osteoarthritis rabbits by HA/hESC-MSC composite were adequately confirmed by the corresponding scores based on the abovementioned staining (Fig. 7e–g). Therefore, in consistent with the in vitro results, HA could reinforce the therapeutic effect of hPSC-MSCs upon osteoarthritis in vivo as well.

Furthermore, based on the aforementioned in vivo parameters, we turned to the principal component analysis (PCA) to determine the potential relationships among the indicated groups (Additional file 5: Figure S5a). We found that the first and second principal component (F1, F2) represented 55.09% and 16.41% of total variability, respectively. Meanwhile, a positive correlation was observed between HA/hESC-MSCs (word labeled in blue) and some variables corresponding to F1 (Mankin score and OARSI macroscopic score) (Fig. 7H, also see Additional file 5: Figure S5a), and in particular, between HA/hESC-MSCs and Sham whereas opposed with the PBS (F2) (Fig. 7H, also see Additional file 5: Figure S5a; dot labeled in blue). Together with the correlation matrix for the parameters, we further confirmed the strongly positive efficacy of HA/hESC-MSCs on osteoarthritis rabbits (value > 0), which was distinguish from the PBS and HA groups as well (value < 0) (Additional file 5: Figure S5a).

## Discussion

hPSC-MSCs including hESC-MSCs and hiPSC-MSCs have been acknowledged as an advantageously alternative source of MSCs in regenerative medicine [21, 22]. However, most of the current procedures are far from satisfaction attribute to untranslatable and cumbersome techniques including mechanical manipulations, viral transduction or enrichment by cell sorting, which also substantially restrict the developmental process and underlying mechanism studies of hPSC-MSCs [23, 27]. Worse still, the similarities and distinctions at transcriptome level between hESC-MSCs and hiPSC-MSCs are largely unknowable. Herein, we took advantage of the programming strategy mediated by combining two small molecules, LLY-507 (a selective inhibitor of EGFR tyrosine kinase and protein-lysine methyltransferase SMYD2) and AZD5153 (a BET/BRD4 bromodomain and E2F/MYC inhibitor), which were involved in epigenetic modification [32–34]. Consistently, the resultant hESC-MSCs and hiPSC-MSCs displayed collectively multifaceted characterizations of MSCs and possessed conservative variations in gene expression profiling as well. Thereafter, we employed the hESC-MSCs for further in vivo study on osteoarthritis treatment and found that the curative effect was preferably enhanced with the aid of HA hydrogel. Taken together, in this study, we introduced a two-step programming procedure for robust and high-efficiency hPSC-MSC generation, which simultaneously provided an applicable and promising strategy for fundamental and clinical research of MSCs.

Despite the consistent and dramatic progress in hPSC-MSC induction, the low efficiency and complexity in manipulation are of the foremost and long-standing concerns before assessment for clinical applications and explorations of the molecular mechanism [21, 27]. In the year of 2005, Barberi and his colleagues firstly reported the induction of hESC-MSCs with a proportion of approximately 5% after 30 days’ induction [35]. Afterwards, Mahmood et al., Yan et al. and Deng et al. utilized the bidimensional monolayer and tree-dimensional EB model for MSC induction from hESC-MSCs and hiPSC-MSCs, respectively [28, 36, 37]. Of the current reports, we have initially reported the establishment of a two-step lentivirus-mediated programming procedure for deriving nearly all of hPSCs into MSCs within two weeks, which was helpful for uncovering the underlying mechanism whereas not suitable for clinical purposes [21]. Recently, we and Wang et al. similarly elevated the efficiency of hPSC-MSC induction to 40–60% by small molecule programming, yet still required additional operations [22, 27]. Herein, with the aid of a small molecule library and optimal combinations as well as in vitro and in vivo analyses, we eventually accomplished the rapid and homogeneous hPSC-MSC manufacture without the abovementioned deficiencies.

The state-of-the-art process and mechanism studies of hPSC-MSCs are pronounced as well [21, 38]. For decades, we and other investigators have indicated the pivotal functions of MSC-associated candidate genes (MSX2, TWIST1, PRAME, EZH2) and signal pathways (JAK/STAT, TGF-β, NF-κB), and in particular, MSX2-TWIST1 axis and IKK/NF-κB-p65 cascade are detailedly and thoroughly dissected by us and Jiang et al., respectively [21, 22]. However, considering the heterogeneity of MSCs caused by organizational distances (e.g., adipose, placenta, bone marrow), original disparities (e.g., mesoderm, endoderm, ectoderm, trophoblast, neural crest) and genetic differences (e.g., hESC-MSCs, hiPSC-MSCs), systematic and meticulous studies on exploring and revealing the all-around biofunctions and regulatory mechanism are the indispensable prerequisites before large-scale clinical applications for disease remodeling and health administration [16, 21, 27, 39]. In consequence, we not only carefully explored the multidimensional and representative signatures and functions as well as the heterogeneous intermediate-stages of the programmed hPSC-MSCs both in vitro and in vivo, but also meticulously dissected the similarities and variations at the transcriptome level, which collectively supplied overwhelming new references and would concurrently benefit the research in the field.

To date, progress in cytobiology and biomaterial technology has prompted the therapeutic applications of tissue engineering for restoring cartilage defects during osteoarthritis [3, 40]. Of the biomaterials, the biocompatible and biodegradable hydrogels could serve as scaffolds and provide structural integrity for morphogenic guidance and cellular organization [40, 41]. For instance, Chung et al. systematically evaluated the curative effect of articular cartilage repair in rats by optimizing the composites formed by hUC-MSCs and different hydrogels including hyaluronic acid (HA), alginate, pluronic, chitosan, and concluded that HA/hUC-MSCs composites resulted in superior cartilage repair and achieved cellular arrangements and collagen organization pattern similar to adjacent uninjured articular cartilage [40]. Thus, in this study, we took advantage of the splendid HA and rabbit osteoarthritis model, and demonstrated the attractive HA/hESC-MSCs composites for use in bone and joint related disease remodeling.

## Conclusions

Overall, we have originally established a two-step procedure for high-efficiency and homologous hPSCs-MSCs generation, which satisfy the multifaceted characterizations of MSCs and display preferable efficacy on osteoarthritis rabbit with HA hydrogel. Above all, the programming strategy would benefit the developmental process and mechanistic investigation as well as heterogeneity of MSCs.

## Methods

### hPSC-MSC differentiation

For hPSC-MSC differentiation, hPSCs were seeded on GFR (Corning, New York, USA)-coated 6-well plate at a density of 2–3 × 10^4^/ml and cultured in E8 medium (with 10 nM Y-27632) for 2 days at 37 °C, 5% CO_2_ [21, 42]. After that, the medium was changed into 3% FBS/DMEM-F12 medium (Gibco, Thermo Fisher Scientific, Massachusetts, USA) with or without the indicated small molecule addition (10 nM) for 9 days. After that, the derived hPSC-MSCs were passaged and cultured with MSC culture medium, which was consist of 10% FBS/DMEM-F12 (Gibco, Thermo Fisher Scientific, Massachusetts, USA), 1% penicillin–streptomycin (Gibco, USA), 1% l-glutamine (Gibco, USA), 10 ng/ml bFGF (PeproTech, USA) and 4 ng/ml EGF (PeproTech, USA). The indicated experiments were conducted for over three independent replications. The detailed information of the detailed procedures and indicated small molecule library (TargetMol, Shanghai, China) was listed in Additional file 6: Additional Procedures and Table S1.

### Multilineage differentiation potential analysis of hPSC-MSCs

The multilineage differentiation potential of hPSC-MSCs towards adipocytes, osteoblasts and chondrocytes were conducted as we described previously [6, 21, 43]. The detailed procedures and primer sequences of the abovementioned genes were listed in Additional file 6: Additional Procedures and Table S2.

### Flow cytometry (FCM) analysis

FCM assay of the immunophenotypes of hPSC-derived cells including hPSC-MSCs were conducted as we reported recently [19, 21, 22]. In brief, the hPSC-derived cells were washed with 1 × PBS for twice and labelled with the conjugated antibodies. After that, the cells were rewashed with 1 × PBS for twice and analyzed by utilizing Canto II flow cytometer (BD Biosciences, USA) and FlowJo 7.6 software (San Carlos, USA). The indicated antibodies were listed in Additional file 6: Table S3.

### Scanning electron microscope (SEM) analysis

The micromorphology of HA hydrogels (HA) and hESC-MSC/HA hydrogel composite (HA/hESC-MSC) were observed by SEM assay with an accelerating voltage of 5 kV as recently reported [44]. Briefly, samples were washed with 1 × PBS, fixed in 4% paraformaldehyde (Sigma-Aldrich, St Louis, USA) for 2 h and thereafter in 1% OsO_4_ for 2 h. Then, they were progressively dehydrated in a series of alcohols after rewashing with 1 × PBS, vacuum-dried, and coated with gold for observation and photograph with the Scanning electron microscope (JEOL JSM-6300F, USA).

### MIA-induced osteoarthritis rabbit model

The MIA-induced osteoarthritis model was conducted as reported with several modification [3, 4, 31]. The histological score was based on the general signs and pathological sections of the knee joints. Ethical approval of animal research was signed by the Ethics Committee of Eye Hospital of Tianjin Medical University (approval number: TJYY2018061114). The detailed procedures were available in Additional file 6: Additional Procedures. All applicable institutional and/or national guidelines for the care and use of animals were followed.

### RNA-SEQ and bioinformatic analyses

The total RNAs for transcriptome analysis were extracted from hESC-MSCs and hiPSC-MSCs and prepared as we recently reported with several modifications [5, 6, 21]. Briefly, 5 × 10^6^ hESC-MSCs or hiPSC-MSCs were used for RNA-SEQ analysis by Novogene (Novogene, Tianjin, China) and the bioinformatic analyses were conducted based on the raw data. The gene expression profiling was available in Additional file 7: Additional Table S4.

### Statistical analysis

All statistical analyses were performed as we reported before [21, 42, 43, 45]. Briefly, the experiments were performed in triplicate for three times and data were acquired as mean values. All data were expressed as mean ± standard deviation (mean ± SD, N = 3 independent experiments). One-way analysis of variance (ANOVA) was used for statistical analysis. P-values were considered significantly when P < 0.05. ****P < 0.00001, ***P < 0.001, **P < 0.01, *P < 0.05; NS, not significant.

## Supplementary Information

**Additional file 1: Figure S1.** FCM assay for hESCs-derived cells after small molecule treatment. (**a**) Flow cytometry (FCM) analysis of hESCs-derived cells cultured with the indicated antibodies (CD73, CD105, CD44, CD31) in 3% FBS/DMEM/F12 ± 10 nM small molecule for 9 days.

**Additional file 2: Figure S2.** hiPSC-MSCs exhibit preferable characteristics in multilineage differentiation. (**a**) The phase contrast images of hiPSC-MSC-derived adipocytes with Oil red O staining. The undifferentiated hiPSC-MSCs were used as negative control. Scale bar = 100 μm. (**b**) Quantitative analysis of the adipogenic-associated genes (*ADIPOQ, PPAR-γ*) in undifferentiated and differentiated hiPSC-MSC-derived adipocytes (mean ± SEM, N = 3). *P < 0.05; **P < 0.01. (**c**) The phase contrast images of hiPSC-MSC-derived osteoblasts with Alizarin Red staining. The undifferentiated hiPSC-MSCs were used as negative control. Scale bar = 100 μm. (**d**) Quantitative analysis of the osteogenic-associated genes (*RUNX2, BGLAP*) in undifferentiated and differentiated hiPSC-MSC-derived osteoblasts (mean ± SEM, N = 3). *P < 0.05; **P < 0.01. (**e**) The phase contrast images of hiPSC-MSC-derived chondrocytes with Alcian Blue staining. The undifferentiated hiPSC-MSCs were used as negative control. Scale bar = 100 μm. (**f**) Quantitative analysis of the chondrogenic-associated genes (*ACAN, SOX9*) in undifferentiated and differentiated hPSC-MSC-derived chondrocytes (mean ± SEM, N = 3). *P < 0.05; **P < 0.01.

**Additional file 3: Figure S3.** qRT-PCR analysis of pluripotency-, germ layer- and MSCs-associated gene expression in hiPSCs and derived cells. (**a**) Statistical analysis of pluripotency-associated gene (*POU5F1*, *SOX2*, *NANOG*) expression in hPSCs during the differentiation process by qRT-PCR assay (mean ± SEM, N = 3). *P < 0.05; **P < 0.01. (**b**) Statistical analysis of germ layer-associated gene (*BRACH*, *GATA2*, *PAX6*) expression in hiPSCs during the differentiation process by qRT-PCR assay (mean ± SEM, N = 3). *, *P* < 0.05, **P < 0.01; NS, not significant. **(c)** Statistical analysis of MSC-associated gene (*NT5E*, *ENG*, *VIM*) expression in hiPSCs during the differentiation process by qRT-PCR assay (mean ± SEM, N = 3). *P < 0.05, **P < 0.01; NS, not significant.

**Additional file 4: Figure S4.** FCM analysis of the immune costimulatory molecules in hESC-MSCs with/without HA hydrogel. (**a**) Representative diagrams of the immune costimulatory molecules (CD40, CD80, CD86) expression in hESC-MSCs with/without HA hydrogel by FCM assay (hESC-MSCs, HA/hESC-MSCs).

**Additional file 5: Figure S5.** Matrix analysis of the relationship of the indicated groups based on multiple parameters of pathological changes and therapeutic effects. (**a**) The correlation matrix scores among the indicated groups (Sham, PBS, HA, hESC-MSCs, HA/hESC-MSCs) based on Pearson analysis as described in the “Methods and Materials” section.

**Additional file 6. **Additional procedures, additional references were listed.

**Additional file 7: Table S4.** The gene expression profiling of hESC-MSCs and hiPSC-MSCs.

## Data Availability

All data generated or analyzed during this study are included in this published article and its supplementary information files. Meanwhile, the datasets used and analyzed during the current study are also available from the corresponding author on reasonable request.

## Abbreviations

hPSCs: Human pluripotent stem cells
hiPSCs: Human induced pluripotent stem cells
hESCs: Human embryonic stem cells
MSCs: Mesenchymal stem/stromal cells
GVHD: Graft-versus-host disease
AMI: Acute myocardial infarction
COVID-19: Corona virus disease 2019
EAE: Experimental autoimmune encephalomyelitis
PBMCs: Peripheral blood mononuclear cells
SEM: Scanning electron microscope
HA: Hyaluronic acid
H&E: Hematoxylin & Eosin
KEGG: Kyoto encyclopedia of genes and genomes
GO: Gene ontology
SNP: Single nucleotide polymorphism
