# Supplementary figures and images for: Two-step generation of mesenchymal stem/stromal cells from human pluripotent stem cells with reinforced efficacy upon osteoarthritis rabbits by HA hydrogel
Source: Cell Biosci. 2021 Jan 6;11:6. doi: 10.1186/s13578-020-00516-x (PMC7787598; doi:10.1186/s13578-020-00516-x)

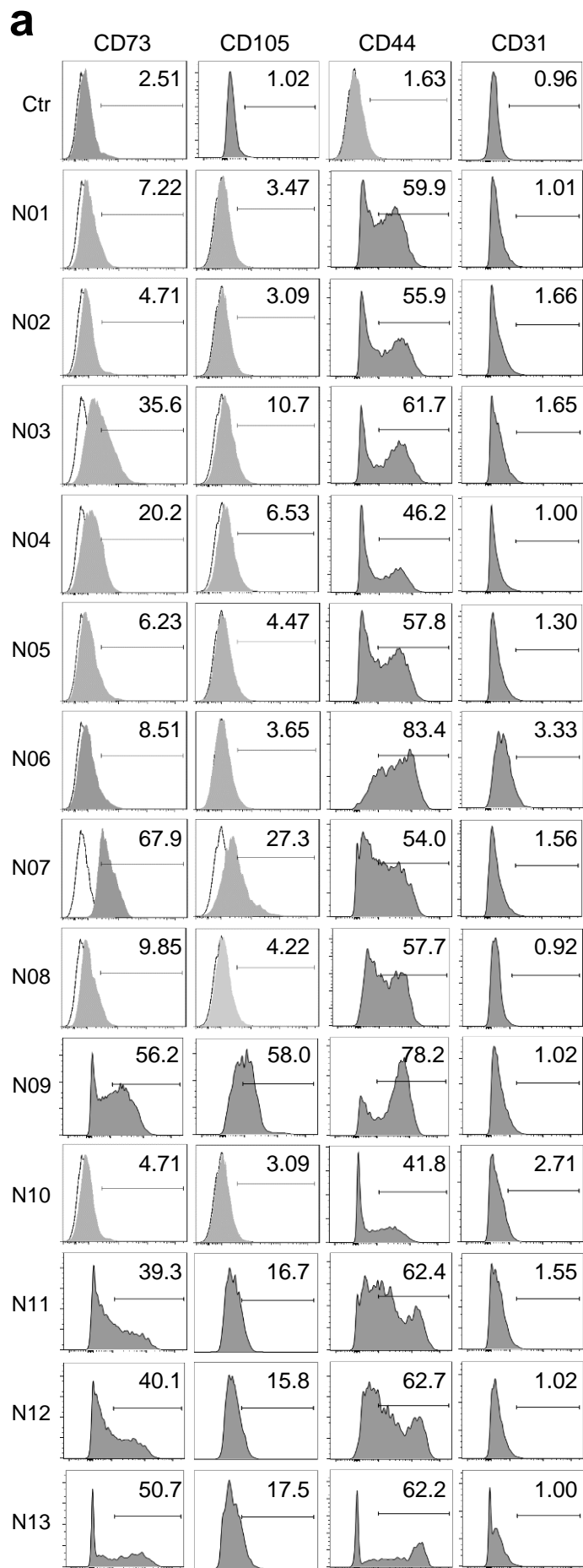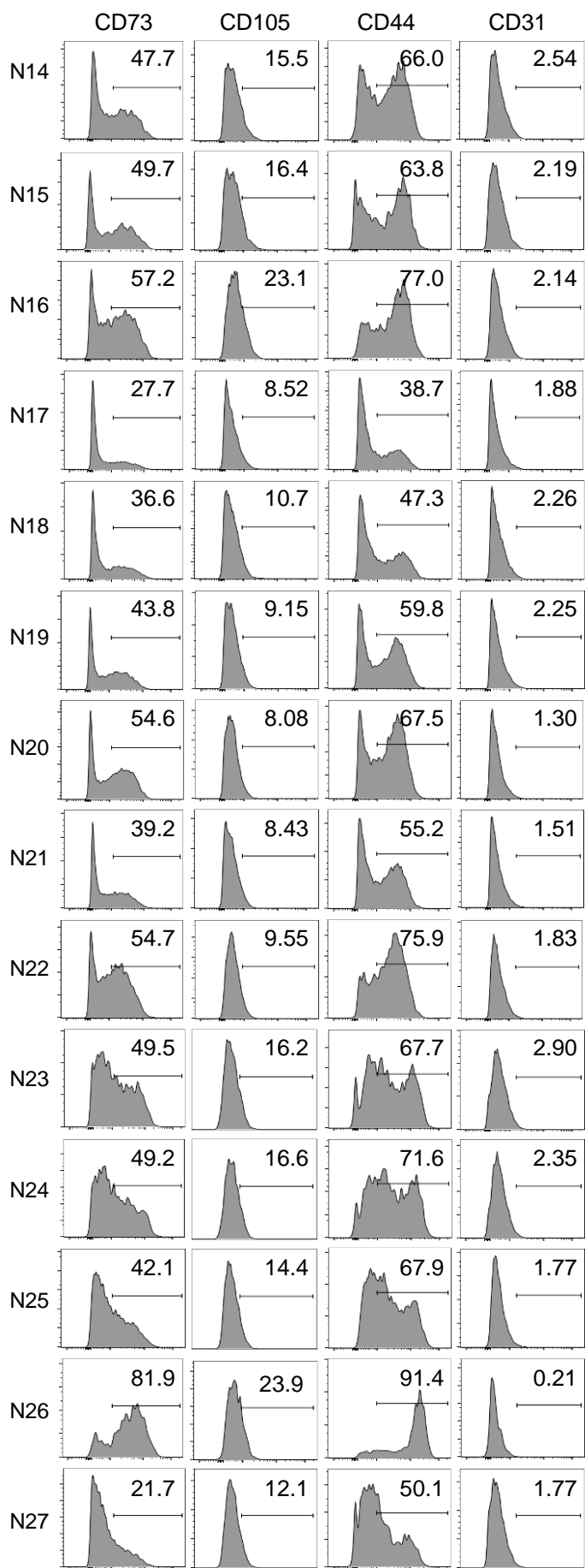

Supplement: Supplementary file 1 — Additional file 1: Figure S1. FCM assay for hESCs-derived cells after small molecule treatment. (a) Flow cytometry (FCM) analysis of hESCs-derived cells cultured with the indicated antibodies (CD73, CD105, CD44, CD31) in 3% FBS/DMEM/F12 ± 10 nM small molecule for 9 days. [file 13578_2020_516_MOESM1_ESM.pdf]

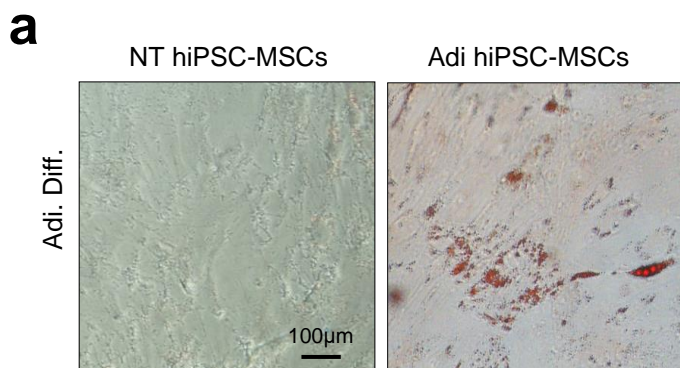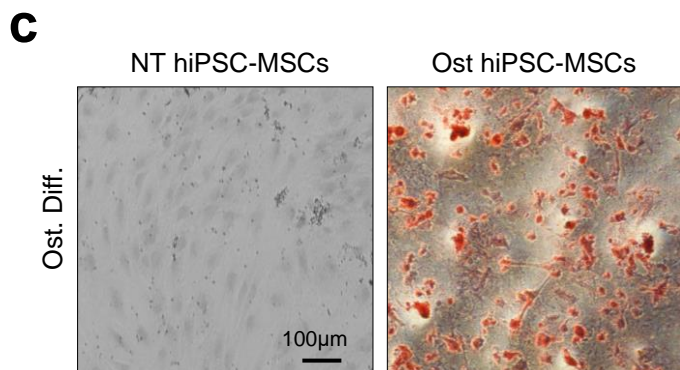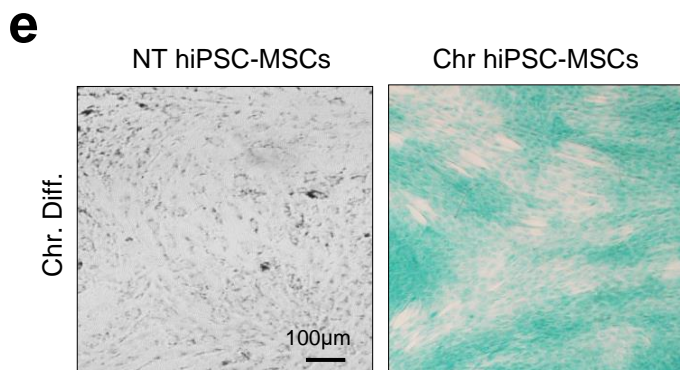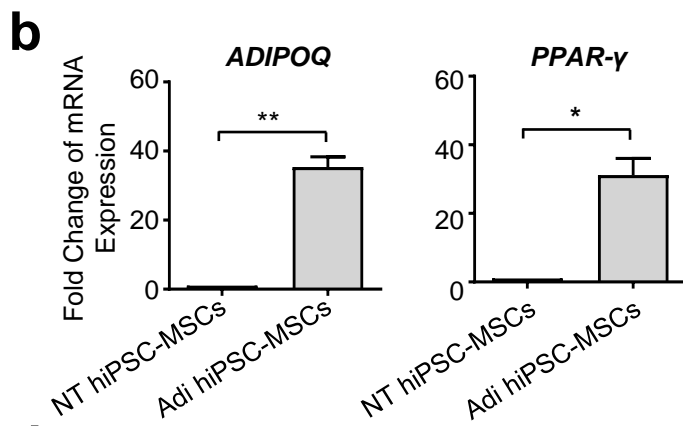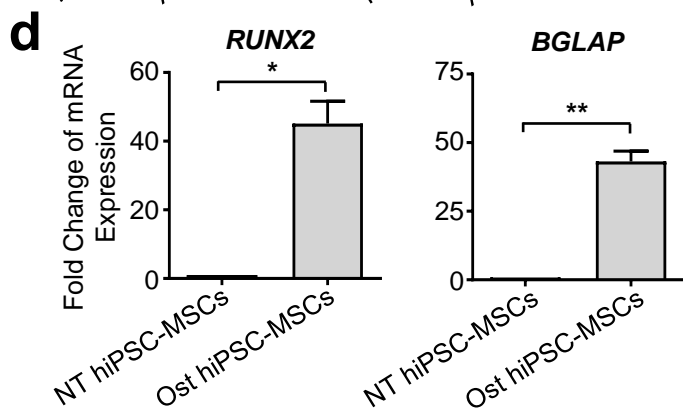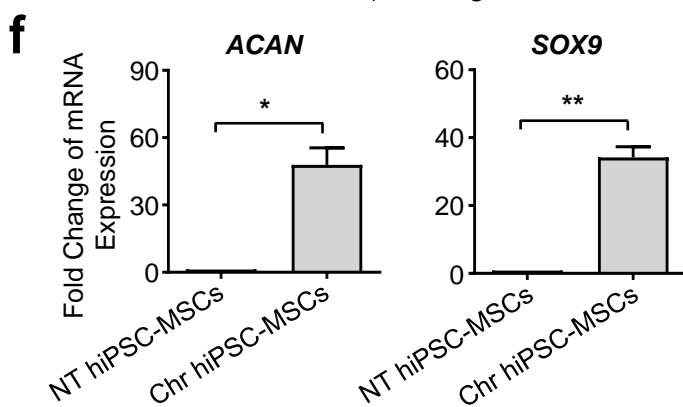

Supplement: Supplementary file 2 — Additional file 2: Figure S2. hiPSC-MSCs exhibit preferable characteristics in multilineage differentiation. (a) The phase contrast images of hiPSC-MSC-derived adipocytes with Oil red O staining. The undifferentiated hiPSC-MSCs were used as negative control. Scale bar = 100 μm. (b) Quantitative analysis of the adipogenic-associated genes (ADIPOQ, PPAR-γ) in undifferentiated and differentiated hiPSC-MSC-derived adipocytes (mean ± SEM, N = 3). *P < 0.05; **P < 0.01. (c) The phase contrast images of hiPSC-MSC-derived osteoblasts with Alizarin Red staining. The undifferentiated hiPSC-MSCs were used as negative control. Scale bar = 100 μm. (d) Quantitative analysis of the osteogenic-associated genes (RUNX2, BGLAP) in undifferentiated and differentiated hiPSC-MSC-derived osteoblasts (mean ± SEM, N = 3). *P < 0.05; **P < 0.01. (e) The phase contrast images of hiPSC-MSC-derived chondrocytes with Alcian Blue staining. The undifferentiated hiPSC-MSCs were used as negative control. Scale bar = 100 μm. (f) Quantitative analysis of the chondrogenic-associated genes (ACAN, SOX9) in undifferentiated and differentiated hPSC-MSC-derived chondrocytes (mean ± SEM, N = 3). *P < 0.05; **P < 0.01. [file 13578_2020_516_MOESM2_ESM.pdf]

**a**

□ hiPSCs    ■ hiPSC-IMCs    ■ hiPSC-MSCs

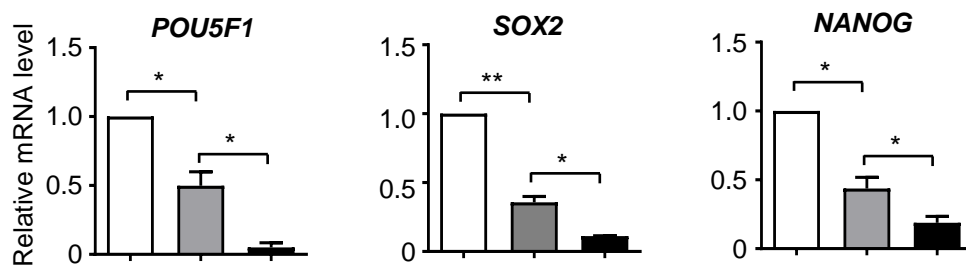**b**

□ hiPSCs    ■ hiPSC-IMCs    ■ hiPSC-MSCs

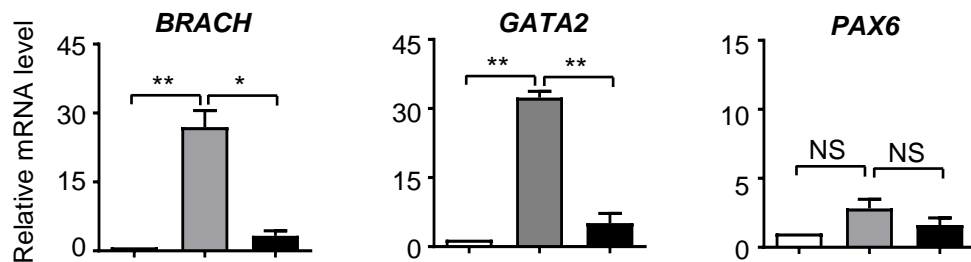**c**

□ hiPSCs    ■ hiPSC-IMCs    ■ hiPSC-MSCs

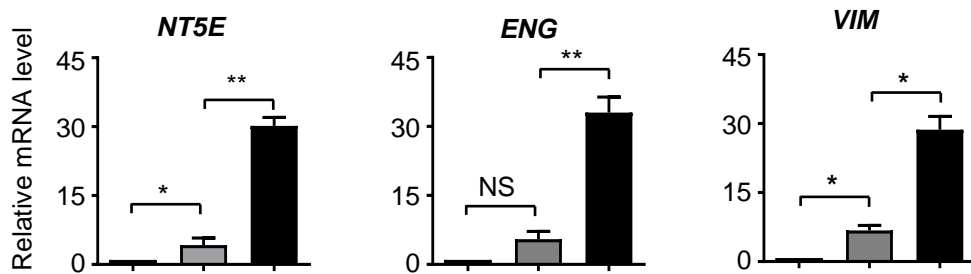

Supplement: Supplementary file 3 — Additional file 3: Figure S3. qRT-PCR analysis of pluripotency-, germ layer- and MSCs-associated gene expression in hiPSCs and derived cells. (a) Statistical analysis of pluripotency-associated gene (POU5F1, SOX2, NANOG) expression in hPSCs during the differentiation process by qRT-PCR assay (mean ± SEM, N = 3). *P < 0.05; **P < 0.01. (b) Statistical analysis of germ layer-associated gene (BRACH, GATA2, PAX6) expression in hiPSCs during the differentiation process by qRT-PCR assay (mean ± SEM, N = 3). *, P < 0.05, **P < 0.01; NS, not significant. (c) Statistical analysis of MSC-associated gene (NT5E, ENG, VIM) expression in hiPSCs during the differentiation process by qRT-PCR assay (mean ± SEM, N = 3). *P < 0.05, **P < 0.01; NS, not significant. [file 13578_2020_516_MOESM3_ESM.pdf]

**a**

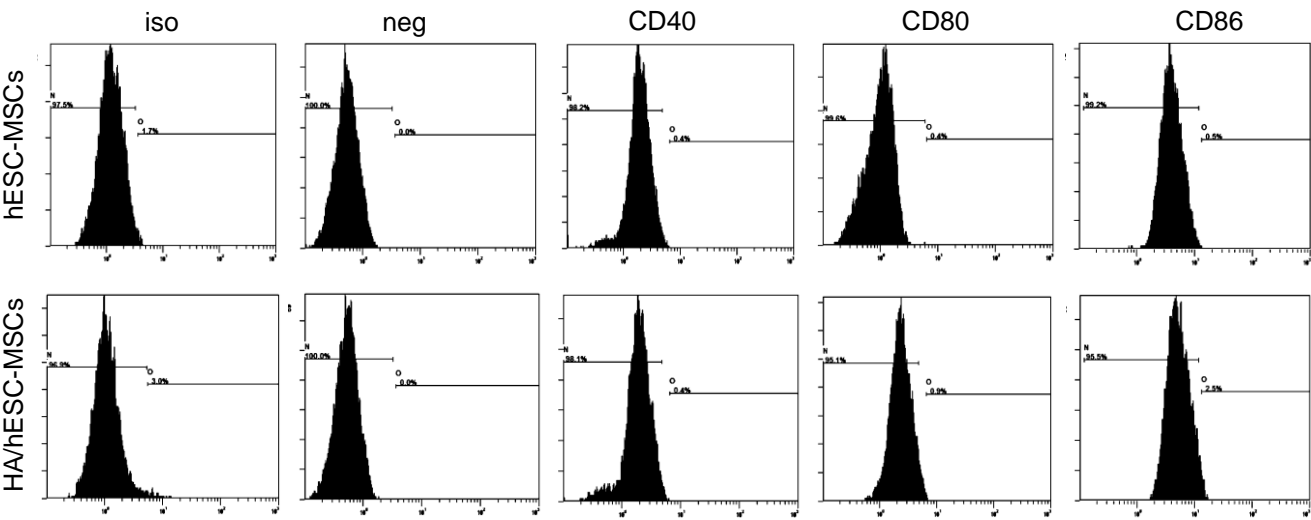

Supplement: Supplementary file 4 — Additional file 4: Figure S4. FCM analysis of the immune costimulatory molecules in hESC-MSCs with/without HA hydrogel. (a) Representative diagrams of the immune costimulatory molecules (CD40, CD80, CD86) expression in hESC-MSCs with/without HA hydrogel by FCM assay (hESC-MSCs, HA/hESC-MSCs). [file 13578_2020_516_MOESM4_ESM.pdf]
