# Supplementary material for: Two-step generation of mesenchymal stem/stromal cells from human pluripotent stem cells with reinforced efficacy upon osteoarthritis rabbits by HA hydrogel
Source: Cell Biosci. 2021 Jan 6;11:6. doi: 10.1186/s13578-020-00516-x (PMC7787598; doi:10.1186/s13578-020-00516-x)
Supplement: Supplementary file 5 — Additional file 5: Figure S5. Matrix analysis of the relationship of the indicated groups based on multiple parameters of pathological changes and therapeutic effects. (a) The correlation matrix scores among the indicated groups (Sham, PBS, HA, hESC-MSCs, HA/hESC-MSCs) based on Pearson analysis as described in the “Methods and Materials” section. [file 13578_2020_516_MOESM5_ESM.pdf]

a

Correlation matrix (Pearson (n))

Variables	Sham	PBS	HA	hESC-MSCs	HA/hESC-MSCs	OARSI macroscopic score	Mankin score (H&E)	Mankin score (Safranin-O)	Mankin score (Alcian Blue)
Sham	**1**	-0.250	-0.250	-0.250	-0.250	0.816	0.632	0.801	0.824
PBS	-0.250	**1**	-0.250	-0.250	-0.250	-0.627	-0.773	-0.584	-0.269
HA	-0.250	-0.250	**1**	-0.250	-0.250	-0.220	-0.304	-0.447	-0.689
hESC-MSCs	-0.250	-0.250	-0.250	**1**	-0.250	-0.251	0.047	-0.031	-0.017
HA/hESC-MSCs	-0.250	-0.250	-0.250	-0.250	**1**	0.282	0.398	0.260	0.151
OARSI macroscopic score	0.816	-0.627	-0.220	-0.251	0.282	**1**	**0.934**	**0.959**	0.831
Mankin score (H&E)	0.632	-0.773	-0.304	0.047	0.398	**0.934**	**1**	**0.956**	0.799
Mankin score (Safranin-O)	0.801	-0.584	-0.447	-0.031	0.260	**0.959**	**0.956**	**1**	**0.932**
Mankin score (Alcian Blue)	0.824	-0.269	-0.689	-0.017	0.151	0.831	0.799	**0.932**	**1**

*** Note:** *Values in red are different from 0 with a significance level (alpha=0.05).*
