# Supplementary material for: Two-step generation of mesenchymal stem/stromal cells from human pluripotent stem cells with reinforced efficacy upon osteoarthritis rabbits by HA hydrogel
Source: Cell Biosci. 2021 Jan 6;11:6. doi: 10.1186/s13578-020-00516-x (PMC7787598; doi:10.1186/s13578-020-00516-x)
Supplement: Supplementary file 6 — Additional file 6. Additional procedures, additional references were listed. [file 13578_2020_516_MOESM6_ESM.docx]

**Additional Information**

**Two-step Generation of Mesenchymal Stem/Stromal Cells from Human Pluripotent Stem Cells with Reinforced Efficacy upon Osteoarthritis Rabbits by HA Hydrogel**

Leisheng Zhang^1-6*^, Yimeng Wei^5^, Ying Chi^5^, Dengke Liu^2^, Sijun Yang^1,6^, Zhongchao Han^4-6^, Zongjin Li^1^

**Additional file 1:**

**Additional Tables: Table S1-S3;**

**Additional Procedures**

**Additional References**

**Additional Tables**

**Table S1. The detailed information of Small molecule library (TargetMol, Shanghai, China) used in this study.**

| **Number** | **Names of small molecules** | **CAS** | **Conc. nMol/L** | **Bioactivity** | **Pathways** | **Receptor** | **Target** |
| --- | --- | --- | --- | --- | --- | --- | --- |
| **N01** | PCI-24781 (Abexinostat) | 783355-60-2 | 10 | PCI-24781 (Abexinostat) is a novel pan-HDAC inhibitor mostly targeting HDAC1 with Ki of 7 nM, modest potent to HDACs 2, 3, 6, and 10 and greater than 40-fold selectivity against HDAC8. Phase 1/2. | Chromatin/Epigenetic; DNA Damage/DNA Repair; NF-Κb | HDAC1; HDAC10; HDAC2; HDAC3/SMRT; HDAC6 | HDAC inhibitor |
| **N02** | EPZ011989 | 1598383-40-4 | 10 | EPZ011989 is a potent, selective orally bioavailable EZH2 inhibitor with Ki < 3 nM for EZH2 wt and EZH2 Y646; 15-fold selectivity over EZH1 and >3000-fold selectivity over other HMTase. | Chromatin/Epigenetic | EZH2 | Histone Methyltransferase inhibitor |
| **N03** | J147 | 1146963-51-0 | 10 | J-147 is a broad spectrum neuroprotective phenyl hydrazide compound(EC50=60-115 nM) with significant neurotrophic properties related to the induction of brain-derived neurotrophic factor (BDNF). | Chromatin/Epigenetic | BDNF | Epigenetic Reader Domain |
| **N04** | UNC1999 | 1431612-23-5 | 10 | UNC1999 is a potent, orally bioavailable and selective inhibitor of EZH2 and EZH1 with IC50 of 2 nM and 45 nM in cell-free assays, respectively, showing >1000-fold selectivity over a broad range of epigenetic and non-epigenetic targets. | Chromatin/Epigenetic | EZH1; EZH2 | Histone Methyltransferase inhibitor |
| **N05** | Fedratinib (SAR302503, TG101348) | 936091-26-8 | 10 | Fedratinib (SAR302503, TG101348) is a selective inhibitor of JAK2 with IC50 of 3 nM in cell-free assays, 35- and 334-fold more selective for JAK2 versus JAK1 and JAK3. Phase 2. | Angiogenesis; Apoptosis; Chromatin/Epigenetic; Tyrosine Kinase/Adaptors; JAK/STAT signaling; Stem Cells | RET; FLT3; JAK2; JAK2 (V617F) | c-RET inhibitor; FLT inhibitor; JAK inhibitor |
| **N06** | CUDC-101 | 1012054-59-9 | 10 | CUDC-101 is a potent multi-targeted inhibitor against HDAC, EGFR and HER2 with IC50 of 4.4 nM, 2.4 nM, and 15.7 nM, and inhibits class I/II HDACs, but not class III, Sir-type HDACs. Phase 1. | Chromatin/Epigenetic; Angiogenesis; DNA Damage/DNA Repair; JAK/STAT signaling; NF-Κb; Tyrosine Kinase/Adaptors | EGFR; HDAC; HDAC1; HDAC10; HDAC2; HDAC3; HDAC4; HDAC5; HDAC6; HDAC7; HDAC8; HDAC9; HER2 | EGFR inhibitor; HDAC inhibitor; HER inhibitor |
| **N07** | Entinostat (MS-275) | 209783-80-2 | 10 | Entinostat (MS-275) strongly inhibits HDAC1 and HDAC3 with IC50 of 0.51 μM and 1.7 μM, compared with HDACs 4, 6, 8, and 10. Phase 3. | Chromatin/Epigenetic; DNA Damage/DNA Repair; NF-Κb | HDAC1; HDAC3 | HDAC inhibitor |
| **N08** | BI 2536 | 755038-02-9 | 10 | BI2536 is a potent Plk1 inhibitor with IC50 of 0.83 nM. It shows 4- and 11-fold greater selectivity against Plk2 and Plk3. Phase 2. | Cell Cycle/Checkpoint; PI3K/Akt/mTOR signaling; Tyrosine Kinase/Adaptors | Met; PI3Kα; PLK1; PLK2; PLK3 | c-Met/HGFR inhibitor; PI3K inhibitor; PLK inhibitor |
| **N09** | LLY-507 | 1793053-37-8 | 10 | LLY-507 is a cell-active, potent, and selective inhibitor of protein-lysine Methyltransferase SMYD2. | Chromatin/Epigenetic | SMYD2 | Histone Methyltransferase inhibitor |
| **N10** | Ginkgolide C | 15291-76-6 | 10 | Ginkgolide C, a natural product, can enhance the cardiac function of rats in the body. | Others | Others | Others |
| **N11** | BI-7273 | 1883429-21-7 | 10 | BI-7273 is a potent, selective, and cell-permeable BRD9 BD Inhibitor. | Chromatin/Epigenetic | BRD7; BRD9 | Epigenetic Reader Domain inhibitor |
| **N12** | CPI-637 | 1884712-47-3 | 10 | CPI-637 is a selective and cell-active benzodiazepinone CBP/EP300 bromodomain inhibitor. | Chromatin/Epigenetic | BRD4; CBP; EP300 | Epigenetic Reader Domain inhibitor |
| **N13** | OTX015 | 202590-98-5 | 10 | OTX015 is a potent BET bromodomain inhibitor with EC50 ranging from 10 to 19 nM for BRD2, BRD3, and BRD4. Phase 1. | Chromatin/Epigenetic | BRDs | Epigenetic Reader Domain inhibitor |
| **N14** | UNC1215 | 1415800-43-9 | 10 | UNC1215 is a potent and selective MBT (malignant brain tumor) antagonist, which binds L3MBTL3 with IC50 of 40 nM and Kd of 120 nM, 50-fold selective versus other members of the human MBT family. | Chromatin/Epigenetic | L3MBTL3; L3MBTL3; L3MBTL3-D274A | Epigenetic Reader Domain antagonist |
| **N15** | EPZ6438 | 1403254-99-8 | 10 | EPZ-6438 is a potent, and selective EZH2 inhibitor with Ki and IC50 of 2.5 nM and 11 nM in cell-free assays, exhibiting a 35-fold selectivity versus EZH1 and >4,500-fold selectivity relative to 14 other HMTs. | Chromatin/Epigenetic | EZH2; EZH2 | Histone Methyltransferase inhibitor |
| **N16** | Panobinostat (LBH589) | 404950-80-7 | 10 | Panobinostat (LBH589) is a novel broad-spectrum HDAC inhibitor with IC50 of 5 nM in a cell-free assay. Phase 3. | Chromatin/Epigenetic; DNA Damage/DNA Repair; NF-Κb | HDAC (MOLT-4 cells); HDAC (Reh cells) | HDAC inhibitor |
| **N17** | GSK126 | 1346574-57-9 | 10 | GSK126 is a potent, highly selective EZH2 methyltransferase inhibitor with IC50 of 9.9 nM, >1000-fold selective for EZH2 over 20 other human methyltransferases. | Chromatin/Epigenetic | EZH2 | Histone Methyltransferase inhibitor |
| **N18** | UNC0379 | 1620401-82-2 | 10 | UNC0379 is a selective, substrate competitive inhibitor of N-lysine methyltransferase SETD8 with IC50 of 7.9 μM, high selectivity over 15 other methyltransferases. | Chromatin/Epigenetic | SETD8 | Histone Methyltransferase inhibitor |
| **N19** | SP2509 | 1423715-09-6 | 10 | SP2509 is a selective histone demethylase LSD1 inhibitor with IC50 of 13 nM, showing no activity against MAO-A, MAO-B, lactate dehydrogenase and glucose oxidase. | Chromatin/Epigenetic | LSD1 | Histone Demethylase inhibitor |
| **N20** | (+)-JQ-1 | 1268524-70-4 | 10 | (+)-JQ1 is a BET bromodomain inhibitor, with IC50 of 77 nM/33 nM for BRD4(1/2) in cell-free assays, binding to all bromodomains of the BET family, but not to bromodomains outside the BET family. | Chromatin/Epigenetic | BRD4 (1); BRD4 (2) | Epigenetic Reader Domain inhibitor |
| **N21** | PFI-3 | 1819363-80-8 | 10 | A small molecule FAK kinase inhibitor, GSK2256098, inhibits growth and survival of pancreatic ductal adenocarcinoma cells. | Chromatin/Epigenetic | SMARCA2; SMARCA4; PB1(5) bromodomains | Epigenetic Reader Domain inhibitor |
| **N22** | UNC669 | 1314241-44-5 | 10 | UNC669 is a potent and selective MBT (malignant brain tumor) inhibitor with IC50 of 6 μM for L3MBTL1, 5- and 11-fold selective over L3MBTL3 and L3MBTL4. | Chromatin/Epigenetic | L3MBTL1; L3MBTL3; L3MBTL4 | Epigenetic Reader Domain antagonist |
| **N23** | CPI-455 | 1628208-23-0 | 10 | CPI-455 is a specific KDM5 inhibitor. | Chromatin/Epigenetic | KDM5A | Histone Demethylase inhibitor |
| **N24** | OICR-9429 | 1801787-56-3 | 10 | OICR-9429 is a potent antagonist of the interaction of WDR5 with peptide regions of MLL and Histone 3 and reduces viability of acute myeloid leukemia cells in vitro. | Angiogenesis; Chromatin/Epigenetic; JAK/STAT signaling; Stem Cells | WDR5 | JAK antagonist |
| **N25** | MS023 | 1831110-54-3 | 10 | MS023 is a potent, selective, and cell-active Type I PRMT inhibitor with IC50 of 30 nM, 119 nM, 83 nM, 4 nM, and 5 nM for PRMT1, PRMT3, PRMT4, PRMT6 and PRMT8, respectively. | Chromatin/Epigenetic | PRMT1; PRMT3; PRMT4; PRMT6; PRMT8 | Histone Methyltransferase inhibitor |
| **N26** | AZD5153 | 1869912-40-2 | 10 | AZD 5153 an orally available, bivalent inhibitor of the bromodomain and extraterminal (BET) protein BRD4 (IC50 = 5 nM). It can simultaneously bind two bromodomains in BRD4, which has been shown to increase antitumor activity in multiple xenograft models of acute myeloid leukemia, multiple myeloma, and diffuse large B cell lymphoma. | Chromatin/Epigenetic | BD1-BRD4; FL-BRD4 | Epigenetic Reader Domain inhibitor |
| **N27** | EED226 | 2083627-02-3 | 10 | EED226 is a potent, selective, and orally bioavailable embryonic ectoderm development (EED) inhibitor with an IC50 of 22 nM. | Chromatin/Epigenetic | EED; PRC2 | DNA Methyltransferase inhibitor; Histone Methyltransferase inhibitor |

**Table S2. Primers used in this study.**

Real-time PCR primer sequences.

| Gene | Forward sequence (5’→3’) | Reverse sequence (3’→5’) |
| --- | --- | --- |
| *ACTIN* | CTCTTCCAGCCTTCCTTCCT | AGCACTGTGTGTTGGCGTACAG |
| *ADIPOQ* | TGGTCCTAAGGGAGACATCG | TGGAATTTACCAGTGGAGCC |
| *PPAR-γ* | GCTGGCCTCCTTGATGAATA | TGTCTTCAATGGGCTTCACA |
| *RUNX2* | CTCACTACCACACCTACCTG | TCAATATGGTCGCCAAACAGATTC |
| *BGLAP* | GGCGCTACCTGTATCAATGG | TCAGCCAACTCGTCACAGTC |
| *ACAN* | CCCCTGCTATTTCATCGACCC | GACACACGGCTCCACTTGAT |
| *SOX9* | AATGGAGCAGCGAAATCAAC | CAGAGAGATTTAGCACACTGATC |
| *POU5F1* | CTTGAATCCCGAATGGAAAGGG | GTGTATATCCCAGGGTGATCCTC |
| *SOX2* | GCCGAGTGGAAACTTTTGTCG | GGCAGCGTGTACTTATCCTTCT |
| *NANOG* | TTTGTGGGCCTGAAGAAAACT | AGGGCTGTCCTGAATAAGCAG |
| *BRACH* | CTGGGTACTCCCAATGGGG | GGTTGGAGAATTGTTCCGATGA |
| *GATA2* | ACTGACGGAGAGCATGAAGAT | CCGGCACATAGGAGGGGTA |
| *PAX6* | ATGTGTGAGTAAAATTCTGGGCA | GCTTACAACTTCTGGAGTCGCTA |
| *NT5E* | GGGCGGAAGGTTCCTGTAG | GAGGAGCCATCCAGATAGACA |
| *ENG* | AGCCCCACAAGTCTTGCAG | GCTAGTGGTATATGTCACCTCGC |
| *VIM* | GAAGAGAACTTTGCCGTTGAAG | GAAGGTGACGAGCCATTT |
| *COL2A1* | TGGACGATCAGGCGAAACC | GCTGCGGATGCTCTCAATCT |
| *AGG* | TCTGTAACCCAGGCTCCAAC | AAATCACCCCTAAAACGGTC |

**Table S3. Antibodies used in this study.**

Antibodies for flow cytometry.

| Antibody |  | Cat. NO. | Source |
| --- | --- | --- | --- |
| Anti-CD44-PE |  | 550989 | BD Pharmigen |
| Anti-human CD73-PE/Cy7 |  | 344009 | BioLegend |
| Anti-human CD90-FITC |  | 555595 | BD Pharmigen |
| Anti-human CD105-APC |  | 800507 | BioLegend |
| Anti-human CD31-PE |  | 560975 | BD Pharmigen |
| Anti-human CD34-PE/Cy7 |  | 343515 | BioLegend |
| Anti-human CD45-APC |  | 560915 | BD Pharmigen |
| Anti-human HLA-DR-PE |  | 560943 | BD Pharmigen |
| Anti-human CD4-Percy5.5 |  | 300530 | BioLegend |
| Anti-human CD8-APC |  | 301014 | Biolegend |
| Anti-human CD8-PE/Cy7 |  | 344712 | Biolegend |
| Anti-human IFNγ-FITC |  | 502506 | Biolegend |
| Anti-human IL-4-PE |  | 12-7049-42 | Invitrogen |
| Anti-human IL17A-APC |  | 17-7149-42 | Invitrogen |
| 594 donkey anti-mouse IgG |  | R37115 | Invitrogen |
| Anti-β-Tubulin I antibody |  | SAB4200715 | Sigma-aldrich |
| Anti-human CD40-FITC |  | CD4001 | Invitrogen |
| Anti-human CD80-FITC |  | MHCD8001 | Invitrogen |
| Anti-human CD86-FITC |  | MHCD8601 | Invitrogen |
| DAPI |  | 62247 | Thermo Scientific™ |

Antibodies for western-blotting assay.

| Name | Company | Catalog | Host | Dilution |
| --- | --- | --- | --- | --- |
| OCT3/4 | Santa Cruz | SC-9081 | Rabbit | 1:1000 |
| SOX2 | Millipore | AB5603 | Rabbit | 1:500 |
| NANOG | Cell Signaling | 3580 | Rabbit | 1:300 |
| α-Tubulin | Abcam | Ab11304 | Mouse | 1:10000 |
| BRACH | Proteintech | 20741-1-AP | Rabbit | 1:300 |
| GATA2 | Proteintech | 11103-1-AP | Rabbit | 1:1000 |
| PAX6 | Proteintech | 12323-1-AP | Rabbit | 1:500 |
| VIM | Proteintech | 10366-1-AP | Rabbit | 1:500 |
| FN1 | Proteintech | 15613-1-AP | Rabbit | 1:300 |

**Additional Procedures**

**hPSCs culture and hPSC-MSC differentiation**

hPSCs including H1 hESCs (WiCell Research Institute, USA) and BC1 hiPSCs (from Cheng Lab) were cultured in E8 medium on Matrigel (BD Biosciences, USA)-coated 6-well plates as we described before with several modification^[^[^1^](#_ENREF_1)^,^ [^2^](#_ENREF_2)^]^. For hPSC-MSC differentiation, hPSCs were seeded on growth factor reduced Matrigel (GFR, Thermo Fisher Scientific, USA)-coated 6-well plate at a density of 2-3×10^4^/ml and cultured in E8 medium (Gibco, Thermo Fisher Scientific, USA) (with 10 nM Y-27632 addition) for 2 days at 37 ℃, 5% CO_2_. After that, the maintenance culture medium was changed into hPSC-MSC induction medium. That is, 3% FBS/DMEM-F12 medium (DMEM/F12 basal medium (Hyclone) supplied with 3% fetal bovine serum (Gibco, Thermo Fisher Scientific, Massachusetts, USA), 1% penicillin-streptomycin (Gibco), 1% L-glutamine (Gibco)) with or without the indicated small molecule addition (10 nM) for 9 days. The abovementioned hPSC-MSC induction medium was changed every two days. Phase contrast images of cells at indicated time points were photographed with a Nikon ElipseTi-U microscope (Nikon, Japan). The detailed information of the indicated small molecule library (TargetMol, Shanghai, China) was listed in Additional Information: Additional Table S1.

**Multilineage differentiation potential analysis of hPSC-MSCs**

The multilineage differentiation potential of hPSC-MSCs towards adipocytes, osteoblasts and chondrocytes were conducted as we described previously^[^[^2-5^](#_ENREF_2)^]^. Briefly, the hPSC-MSCs were seeded at a density of 2×10^4^ cells/cm^2^ in DMEM/F12 medium (Hyclone, USA) containing 10% FBS (Gibco, USA), 1% penicillin-streptomycin (Gibco, USA), 1% L-glutamine (Gibco, USA). When cells reached 80% confluence, the medium was changed into adipogenic differentiation medium (MesenCult™ Adipogenic Differentiation Kit, Stem Cell Technologies, USA), osteogenic differentiation medium (MesenCult™ Osteogenic Differentiation Kit, Stem Cell Technologies, USA) or chondrogenic differentiation medium (MesenCult™-ACF Chondrogenic Differentiation Kit, Stem Cell Technologies, USA), respectively. The morphology of hPSC-MSC-derived adipocytes, osteoblasts or chondrocytes were shown by Oil red O staining, Alizarin red staining or Alcian blue staining after 18 days’ differentiation and photographed with a Nikon ElipseTi-U microscope (Nikon, Japan), respectively. The undifferentiated hPSC-MSCs were used as negative controls. For quantitative analyses of adipogenic-, osteogenic- or chondrogenic-associated gene expression, the differentiatiated hPSC-MSCs at day 18 were washed with 1×PBS and lysed with TRIzol reagent (ThermoFisher, USA) for total RNA extraction and expression detection. The primer sequences of the abovementioned genes were listed in Additional Information: Additional Table S2.

**Chromosome karyotyping**

With the aid of a G-banding technique, the genomic stability of hPSC-MSCs was monitored by using karyotypic analysis as we recently reported^[^[^4^](#_ENREF_4)^,^ [^6^](#_ENREF_6)^]^. Briefly, the hPSC-MSCs in metaphase were photographed under an Olympus DA71 microscope (Tokyo, Japan) and the chromosome karyotyping of the cells were analyzed as well.

**Population doubling (Pd) assay**

The Pd analysis of derived hPSC-MSCs were displayed as we recently reported^[^[^6^](#_ENREF_6)^]^. In details, 1×10^4^ hPSC-MSCs (H1 hESC-MSCs, BC1 hiPSC-MSCs) were collected and seeded into 3cm^2^ plates. hPSC-MSCs were cultured for 7 days and the culture medium was changed every 3.5 days. Pd number (PDN) was calculated by utilizing the formula PDN=logN/N_0_×3.31, Pd time (PDT) was calculated as following: PDT= (t-t_0_)×log2/log(N_t_-N_0_). N_0_ and N_t_ represents the initially seeded and harvested cell number, respectively. t-t_0_ represents the duration of cell culture.

**cDNA synthesis and real-time PCR (qRT-PCR) assay**

The total mRNA was isolated with TRIzol reagent (Thermo Fisher Scientific Massachusetts, USA) from hPSC-MSCs or hPSC-MSC-derived cells as we reported before^[^[^4^](#_ENREF_4)^,^ [^7^](#_ENREF_7)^]^. Then, cDNA was synthesized by conducting reverse transcription polymerase chain reaction (RT-PCR) with the TransScript Fly First-Strand cDNA Synthesis SuperMix Kit (Transgen Biotech, Beijing, China). The expressive abundances of the indicated genes were detected by utilizing the SYBR Green PCR Master Mix (Qiagen, Germany) together with ABI PRISM 7900 (Applied Biosystems, Thermo Fisher Scientific Massachusetts, USA). The primer sequences were listed in Additional Information: Additional Table S2.

**Western-blotting analysis**

The expressive abundances of the indicated genes at the protein level were quantified by western-blotting analysis as we conducted previously^[^[^2^](#_ENREF_2)^,^ [^8^](#_ENREF_8)^]^. In details, the total proteins were collected from hPSCs and hPSC-derived cells with laemmli sample buffer (Bio-Rad, USA). After protein inactivation, the pretreated samples were turned to SDS-PAGE gel electrophoresis and blot with PVDF membrane (Life Sciences, USA). Thereafter, the membrane was labeled with primary (Cell Signaling, Abcam, USA) and HRP-conjugated secondary (GE Healthcare, USA) antibody after blocking with 5% milk (BD Biosciences), respectively. Finally, the membrane was developed with ECL Detection Reagent (ThermoFisher Scientific Massachusetts, USA) and Super-signal West Pico Chemiluminescent Substrate (Prierce, USA). The indicated antibodies were listed in Additional Information: Additional Table S3.

***In vitro* wound healing assay**

The migration capacity of hPSC-MSCs was evaluated by conducting wound healing analysis^[^[^6^](#_ENREF_6)^,^ [^9^](#_ENREF_9)^]^. In details, the scratch wound was realized by a 10 μl pipette tip when hPSC-MSCs reached 80% confluence and culture medium was changed into 2% FBS/DMEM-F12 medium (Gibco, Thermo Fisher Scientific, Massachusetts, USA). Cell migration was calculated based on the gap area measured by Image J software (NIH, USA) as following: % of area repopulation = 1- clear area of t_n_/clear area of t_0_ (t_0_ represents the initial time point, t_n_ represents the end time point).

**Colony-forming unit-fibroblast (CFU-F) analysis**

The CFU-F analysis of hPSC-MSCs was performed as we previously reported with minimal modifications^[^[^2^](#_ENREF_2)^,^ [^6^](#_ENREF_6)^]^. Briefly, hPSC-MSCs were seeded into 5 cm dishes at a density of 2×10^3^/dish with culture medium replacement every 3 days. Two weeks later, the CFU-Fs formed by the seeded hPSC-MSCs were fixed with 4% paraformaldehyde (PFA, Sigma-Aldrich, St Louis, USA) and followed by dying with 0.5% crystal violet staining solution (Solarbio, Beijing, China). The standard CFU-F colonies with more than 30 cells were counted and calculated.

**Co-culture of PBMCs with hPSC-MSCs**

The inhibitory effect of hPSC-MSCs on lymphocyte proliferation was verified by co-culture of CD4^+^ T cells from PBMCs with hPSC-MSCs as we described recently^[^[^6^](#_ENREF_6)^,^ [^10^](#_ENREF_10)^]^. Briefly, 1×10^6^ CD4^+^ T cells from PBMCs were cocultured with or without 2×10^5^ hPSC-MSCs. 3 days later, the CD4^+^ T cells were turned to FACS Canto Ⅱ (BD Biosciences, USA) for The Th1, Th2 or Th17 cells analyses. The detailed procedures and indicated antibodies were listed in Additional Information: Additional Procedures and Table S3.

**Immunofluorescence staining**

The immunofluorescence staining on hPSC-MSCs were performed as we recently described with several modification^[^[^4^](#_ENREF_4)^,^ [^6^](#_ENREF_6)^,^ [^7^](#_ENREF_7)^]^. In details, hPSC-MSCs or hESC-MSC/HA hydrogel composite (HA+hESC-MSC) were washed with 1×PBS for twice and fixed with 4% (w/v) paraformaldehyde solution (Sigma-Aldrich, St Louis, USA) for 10 min. For F-actin cytoskeleton analysis, the cells were permeabilizated with 0.1% Triton X-100 solution (Sigma-Aldrich, St Louis, USA) for 20 min at room temperature (RT), and stained with Alexa Fluor 488 Phalloїdin (Invitrogen, Thermo Fisher Scientific Massachusetts, USA) for 30 min. Finally, after washing twice with DMEM without phenol red, the immunofluorescent images were captured with a laser scanning confocal microscope (Leica TCS SP2, Microsystem Inc, Germany).

**Microtubule formation analysis**

Microtubule formation analysis of hPSC-MSCs was conducted as reported with several modifications^[^[^6^](#_ENREF_6)^,^ [^11^](#_ENREF_11)^]^. Briefly, hPSC-MSCs were seeded into a Matrigel (BD Biosciences, USA)-coated 48-well plate at a density of 2×10^4^/well and photographed at the indicated time points. The Image J software (NIH, USA) was used to qualify the total tube number and length of capillary-like structures.

**Co-culture of PBMCs with hPSC-MSCs**

The inhibitory effect of hPSC-MSCs on lymphocyte proliferation was verified by co-culture of CD4^+^ T cells from human peripheral blood mononuclear cells (PBMCs) with hPSC-MSCs as we described recently^[^[^6^](#_ENREF_6)^,^ [^10^](#_ENREF_10)^]^. In details, the PBMCs were isolated by utilizing standard Ficoll (Solarbio, Beijing, China) density gradient centrifugation, while CD4^+^ T cells were further enriched with magnetic MicroBead kits (Miltenyi Biotec, USA) from PBMCs as we reported before^[^[^12^](#_ENREF_12)^]^. Then, 1×10^6^ CD4^+^ T cells were cocultured with or without 2×10^5^ hPSC-MSCs in IMDM basal medium (Hyclone, Australia) containing 10% FBS, 2 μM monensin (BD Biosciences, USA), 1 μg/ml Ionomycin (Sigma) and 100 ng/ml Phorbol-12-myristate-13-acetate (PMA) (Sigma-Aldrich, St Louis, USA) at 37°C, 5% CO_2_. 3 days later, the CD4^+^ T cells were labeled with conjugated antibodies and detected by FACS Canto Ⅱ (BD Biosciences, USA). The Th1 or Th2 cells were labeled with antibodies against CD4, IFN-γ and IL-4, while the Th17 cells were labeled with CD4, IL-4 and IL17. The indicated antibodies were listed in Additional information, Table S3.

**Preparation for** **hESCs and HA hydrogel Composite**

hESC-MSCs was thoroughly mixed with 1% HA to create hESC-MSCs and HA hydrogel composites. Inject the cells in the same way as the previous of MIA, repeat the above operation were performed at day7, twice in total.

**Ethics approval**

The experiment on rabbits followed the internationally recognized guidelines. Meanwhile, ethical approval of animal research was signed by the Ethics Committee of Eye Hospital of Tianjin Medical University (approval number: TJYY2018061114).

**Animals**

Fifteen healthy New Zealand white male rabbits (weight, 2-2.25 kg) were used in this study. All animals were obtained one week before the experiment and were raised in the same environment. Rabbits are kept in the experimental animal room of Chinese Academy of Medical Sciences & Peking Union Medical College Institute of Radiation Medicine. All procedures and experimental protocols were reviewed and approved by the Institutional Animal Care and Use Committee at our institution. This study followed the National Institutes of Health guidelines regarding the care and use of laboratory animals.

**MIA-induced OA rabbit model**

The abovementioned rabbits were divided into five experimental groups, three rabbits were served as control for detecting the histologically normal appearance of the Knee joint. After one week, rabbits were anesthetized with a 3.5% isoflurane to oxygen mixture. The hairs around the knee joint were removed by utilizing an electric hair clipper, the leg skin was sterilized with 75% ethyl alcohol. 100 μl doses of 2% concentrations of sodium iodoacetate (MIA, Sinopharm Group Tianjin Co., Ltd, China) were injected into the joints of rabbits in each group by using an arthrocentesis technique. Then, a needle was inserted vertically to penetrate the skin and turned distally for insertion into the articular cavity until a distinct loss of resistance was felt. Repeat the above operation at day 7 of the OA rabbit model, that is, two times in total. All applicable institutional and/or national guideline for the care and use of animals were followed.

**Cartilage histology**

In preparation for histologic and immunohistochemistry analyses, the joints of the rabbits in the abovementioned groups were fixed in 10% formaldehyde. After that, the fixed joints were decalcified in ethylenediaminetetraacetic acid disodium salt solution, and embedded in paraffin wax. Paraffin-embedded sections (4 μm in thickness) were prepared and then followed by deparaffinization. For histological analysis, multidimensional experiments were practiced including hematoxylin and eosin (H & E) staining, Safranin-O staining and Alcian blue staining.

**The Study Scheme**

Rabbits were injected every week for three weeks with a material according to an experimental group after the second week of the OA model. At the ninth week of the OA model, all the rabbits were euthanized, and stifle joint samples were collected for further evaluation.

**Routine tests & Macroscopic examination**

During the osteoarthritis (OA) model and treatment, we measured the variations of rabbit body weight and joint circumference. The surface of distal femur was exposed and examined macroscopically. To assess the histological cartilage remediation efficiency, sections were analyzed according to a modified Mankin’s score. The nature of the predominant tissue, structural characteristics (surface regularity, structural integrity, thickness, bonding to the adjacent cartilage), freedom from cellular changes of degeneration (hypocellularity, chondrocyte clustering), and freedom from degenerative changes in adjacent cartilage were collectively assessed as well. The grade of osteoarthritic variations of all femoral condyles was analyzed by the well-established Mankin’s score grading for osteoarthritis cartilage histopathology. All samples were evaluated independently by two observers who were totally blinded to sample information.

**Principal Component Analysis (PCA)**

The effects of treatment groups and their correlation with the others parameters were determined statistically by means of the Principal Component Analysis (PCA) with the XLSTAT software (Addinsoft, New York, USA).

**Additional References**

[1] Wu Q, Zhang L, Su P, et al. Msx2 mediates entry of human pluripotent stem cells into mesendoderm by simultaneously suppressing sox2 and activating nodal signaling. Cell Res, 2015, 25: 1314-1332

[2] Zhang L, Wang H, Liu C, et al. Msx2 initiates and accelerates mesenchymal stem/stromal cell specification of hpscs by regulating twist1 and prame. Stem Cell Reports, 2018, 11: 497-513

[3] Zhang X, Yang Y, Zhang L, et al. Mesenchymal stromal cells as vehicles of tetravalent bispecific tandab (cd3/cd19) for the treatment of b cell lymphoma combined with ido pathway inhibitor d-1-methyl-tryptophan. J Hematol Oncol, 2017, 10: 56

[4] Wei Y, Zhang L, Chi Y, et al. High-efficient generation of vcam-1(+) mesenchymal stem cells with multidimensional superiorities in signatures and efficacy on aplastic anaemia mice. Cell Prolif, 2020, e12862

[5] Wang L, Zhang L, Liang X, et al. Adipose tissue-derived stem cells from type 2 diabetics reveal conservative alterations in multidimensional characteristics. Int J Stem Cells, 2020,

[6] Huo J, Zhang L, Ren X, et al. Multifaceted characterization of the signatures and efficacy of mesenchymal stem/stromal cells in acquired aplastic anemia. Stem Cell Res Ther, 2020, 11: 59

[7] Wei Y, Hou H, Zhang L, et al. Jnki- and dac-programmed mesenchymal stem/stromal cells from hescs facilitate hematopoiesis and alleviate hind limb ischemia. Stem Cell Res Ther, 2019, 10: 186

[8] Zhang L, Liu C, Wang H, et al. Thrombopoietin knock-in augments platelet generation from human embryonic stem cells. Stem Cell Res Ther, 2018, 9: 194

[9] El Moshy S, Radwan IA, Rady D, et al. Dental stem cell-derived secretome/conditioned medium: The future for regenerative therapeutic applications. Stem Cells Int, 2020, 2020: 7593402

[10] Zhao Q, Zhang L, Wei Y, et al. Systematic comparison of huc-mscs at various passages reveals the variations of signatures and therapeutic effect on acute graft-versus-host disease. Stem Cell Res Ther, 2019, 10: 354

[11] Du W, Li X, Chi Y, et al. Vcam-1+ placenta chorionic villi-derived mesenchymal stem cells display potent pro-angiogenic activity. Stem Cell Res Ther, 2016, 7: 49

[12] Zhang W, Liu C, Wu D, et al. Decitabine improves platelet recovery by down-regulating il-8 level in mds/aml patients with thrombocytopenia. Blood Cells Mol Dis, 2019, 76: 66-71
